# Reduced reticulum–mitochondria Ca^2+^ transfer is an early and reversible trigger of mitochondrial dysfunctions in diabetic cardiomyopathy

**DOI:** 10.1007/s00395-020-00835-7

**Published:** 2020-11-30

**Authors:** Maya Dia, Ludovic Gomez, Helene Thibault, Nolwenn Tessier, Christelle Leon, Christophe Chouabe, Sylvie Ducreux, Noelle Gallo-Bona, Emily Tubbs, Nadia Bendridi, Stephanie Chanon, Aymeric Leray, Lucid Belmudes, Yohann Couté, Mazen Kurdi, Michel Ovize, Jennifer Rieusset, Melanie Paillard

**Affiliations:** 1 Laboratoire CarMeN-Équipe 5 Cardioprotection, INSERM, INRA, Université Claude Bernard Lyon-1, INSA-Lyon, Univ-Lyon, U1060 CARMEN, Equipe 5- Cardioprotection, Groupement Hospitalier Est, Bâtiment B13, 59 boulevard Pinel, 69500 Bron, France; 2 Laboratory of Experimental and Clinical Pharmacology, Faculty of Sciences, Lebanese University-Beirut, Beirut, Lebanon; 3 Laboratoire CarMeN-Équipe 3, INSERM, INRA, Université Claude Bernard Lyon-1, INSA Lyon, Univ-Lyon, 69921 Oullins, France; 4 Laboratoire Interdisciplinaire Carnot de Bourgogne, UMR 6303 CNRS, Université de Bourgogne Franche Comté, 21078 Dijon, France; 5 Univ. Grenoble Alpes, CEA, Inserm, IRIG, BGE, 38000 Grenoble, France; 6 IHU OPERA, Hospices Civils de Lyon, 69500 Bron, France

**Keywords:** Diabetic cardiomyopathy, Ca^2+^ flux, Metabolic syndrome disease, Protein database, Mitochondria-associated membranes MAM, Proteomic analysis of cardiac MAM proteome

## Abstract

Type 2 diabetic cardiomyopathy features Ca^2+^ signaling abnormalities, notably an altered mitochondrial Ca^2+^ handling. We here aimed to study if it might be due to a dysregulation of either the whole Ca^2+^ homeostasis, the reticulum–mitochondrial Ca^2+^ coupling, and/or the mitochondrial Ca^2+^ entry through the uniporter. Following a 16-week high-fat high-sucrose diet (HFHSD), mice developed cardiac insulin resistance, fibrosis, hypertrophy, lipid accumulation, and diastolic dysfunction when compared to standard diet. Ultrastructural and proteomic analyses of cardiac reticulum–mitochondria interface revealed tighter interactions not compatible with Ca^2+^ transport in HFHSD cardiomyocytes. Intramyocardial adenoviral injections of Ca^2+^ sensors were performed to measure Ca^2+^ fluxes in freshly isolated adult cardiomyocytes and to analyze the direct effects of in vivo type 2 diabetes on cardiomyocyte function. HFHSD resulted in a decreased IP3R–VDAC interaction and a reduced IP3-stimulated Ca^2+^ transfer to mitochondria, with no changes in reticular Ca^2+^ level, cytosolic Ca^2+^ transients, and mitochondrial Ca^2+^ uniporter function. Disruption of organelle Ca^2+^ exchange was associated with decreased mitochondrial bioenergetics and reduced cell contraction, which was rescued by an adenovirus-mediated expression of a reticulum-mitochondria linker. An 8-week diet reversal was able to restore cardiac insulin signaling, Ca^2+^ transfer, and cardiac function in HFHSD mice. Therefore, our study demonstrates that the reticulum–mitochondria Ca^2+^ miscoupling may play an early and reversible role in the development of diabetic cardiomyopathy by disrupting primarily the mitochondrial bioenergetics. A diet reversal, by counteracting the MAM-induced mitochondrial Ca^2+^ dysfunction, might contribute to restore normal cardiac function and prevent the exacerbation of diabetic cardiomyopathy.

**Electronic supplementary material:**

The online version of this article (10.1007/s00395-020-00835-7) contains supplementary material, which is available to authorized users.

## Introduction

With the current alarming progression of type 2 diabetes (T2D), diabetic cardiomyopathy (DCM) is nowadays recognized as a major cardiovascular complication with a four-to-fivefold increase in the risk of heart failure for diabetic patients [26, 28]. Hyperglycemia, insulin resistance, and hyperinsulinemia result in cardiac hypertrophy and fibrosis that alter diastolic function [5, 24, 25]. The excitation–contraction coupling and the mitochondria-dependent energy-supply balance, which require fine-tuned calcium (Ca^2+^) dynamics, are potential contributors of DCM progression [4, 20, 24]. In the diabetic cardiomyocyte, a reduced mitochondrial Ca^2+^ uptake [16, 20] contributes to a decreased ATP production [34] and favors reactive oxygen species (ROS) production [71]. Mitochondrial Ca^2+^ mishandling can be considered as a key player of cardiomyocyte dysfunction in DCM. However, the precise mechanisms behind the disruption of mitochondrial Ca^2+^ homeostasis during DCM progression remain poorly known.

In the normal heart, mitochondria and reticulum are organized as a complex network encompassing micro-domains named mitochondria-associated membranes (MAM) that create a hotspot for Ca^2+^ fluxes from reticulum to mitochondria. Ca^2+^ flows through a molecular machinery including first the IP3R/Grp75/VDAC Ca^2+^ channeling and tethering complex [31, 58], and then the mitochondrial Ca^2+^ uniporter. The mitochondrial uniporter that acts as a gatekeeper of Ca^2+^ entry into the mitochondrial matrix is composed of the pore-forming subunit MCU [3, 10], the regulators MICU1/2 [8, 46], and the bridging protein EMRE [53].

In type 2 diabetes, disrupted MAM interface or function has been mostly studied in the main insulin target tissues (i.e., liver and skeletal muscle) as well as in pancreatic beta cells [12, 49, 50, 65], but not in the heart. A decreased mitochondrial Ca^2+^ uptake has been reported in T2D-induced DCM [24], but its mechanism and the ensuing mitochondrial dysfunction have not been elucidated.

In the present study, we questioned whether structural or functional alteration of either the IP3R-Grp75-VDAC channeling complex or the mitochondrial Ca^2+^ uniporter might be involved in the development of mitochondrial Ca^2+^ mishandling during T2D-induced DCM.

To this end, we used a high-fat high-sucrose diet (HFHSD)-induced obesogenic T2D mouse model that displayed cardiac insulin resistance after 16 weeks of the diet. Ultrastructural study and quantitative proteomics of the cardiac MAM interface revealed an altered thickness and protein composition of the HFHSD MAM toward downregulated Ca^2+^ transport process. A reduced formation of the IP3R/Grp75/VDAC Ca^2+^ channeling complexes with a decreased IP3-stimulated Ca^2+^ transfer to mitochondria was measured in the HFHSD cardiomyocyte. This MAM-related mitochondrial Ca^2+^ mishandling disrupted the mitochondrial bioenergetics production and cardiomyocyte contraction, which could be restored by the expression of a MAM linker. At the heart level, these alterations resulted in cardiac hypertrophy and diastolic dysfunction. Importantly, switching back to standard diet (SD) reestablished normal cardiac insulin signaling, MAM functional Ca^2+^ coupling, and the IP3-driven Ca^2+^ transfer to mitochondria, and resulted in the recovery of a normal cardiac function, proving the reversibility of these alterations.

## Materials and methods

### Animal studies

All animal procedures performed conform to the guidelines from Directive 2010/63/EU of the European Parliament on the protection of animals used for scientific purposes. The animal protocol used in this study was approved by the institutional animal research committee from Université Claude Bernard Lyon 1 and the French ministry (#15,627–2018062118508398). Male C57BL/6JOlaHsd mice (4 weeks old) were purchased from ENVIGO and were acclimated to the animal facility for 1 week before the study. Five-week-old mice were randomly assigned to the high-fat high-sucrose diet group (HFHSD: 260HF U8978 version 19, from SAFE: 20% proteins, 36% lipids, and 35% carbohydrates) or to the standard diet group (SD: LASQC diet Rod16-A, Genobios: 16.9% proteins, 4.3% lipids) for up to 24 weeks. A total of 204 mice were used for this study. Glucose and insulin tolerance tests were performed after 6 h of fasting. Glucose (2 mg/g body weight) or insulin (0.75 mU/g body weight) was injected intraperitoneally, and blood glucose levels were monitored using a glucometer at the indicated time points. For testing the in vivo cardiac insulin signaling, 6 h-fasted mice were injected intraperitoneally with insulin (10 mU/g). Fifteen minutes after the insulin injection, the mouse was euthanized by cervical dislocation, and then, the heart was removed and frozen in liquid nitrogen and kept at -80 °C until processing.

### Heart subcellular fractionation

Mice were euthanized by cervical dislocation, and then, hearts were extracted and placed in Starting Buffer (SB: 225 mM mannitol, 75 mM sucrose, and 30 mM Tris HCl, pH 7.4). Atria were removed and heart tissues were minced by scissors, followed by a homogenization using a teflon–glass grinder (15–20 strokes) with 4 ml of IB1 buffer (225 mM mannitol, 75 mM sucrose, 0.5% BSA, 0,5 mM EGTA, and 30 mM Tris HCl) per heart. The homogenates were centrifuged twice at 740 *g*/5 min/4 °C. Then, supernatants were collected and centrifuged at 9000 *g*/10 min/4 °C. The pellets were gently resuspended in IB2 buffer (225 mM mannitol, 75 mM sucrose, 0.5% BSA, and 30 mM Tris HCl, pH 7.4) and centrifuged at 10000 g/10 min/4 °C, while supernatants were preserved as cytosolic fractions. Then, pellets were resuspended again in SB to wash out the BSA and EGTA, followed by centrifugation at 10,000 *g*/10 min/4 °C. Afterwards, the pellets of crude mitochondria were resuspended in Mitochondria Resuspending Buffer (MRB: 250 mM Mannitol, 5 mM HEPES, 0.5 mM EGTA, pH 7.4). To purify the mitochondria and obtain mitochondria-associated membranes (MAMs), separation was done using a Percoll density gradient [225 mM Mannitol, 25 mM HEPES, 1 mM EGTA, and 30% Percoll (Sigma-Aldrich P1664)] in 14 ml thin-wall Beckman ultracentrifuge tubes, under 95,000 *g*/30 min/4 °C ultracentrifugation. Purified mitochondria and MAMs, localized at the bottom of the tube as a dense band and in the middle as a round layer, respectively, were collected and washed by MRB at a centrifugation of 6300*g* /10 min/4 °C, and then, MAMs were re-centrifuged in polycarbonate tubes using the ultracentrifuge at 100,000 *g*/1 h/4 °C. A ring at the bottom of the tubes was collected, being the MAMs. For ER and cytosolic fractions, the cytosolic fractions were centrifuged at 20,000 *g*/30 min/4 °C to remove potential remaining mitochondria. Next, the supernatants were collected and re-centrifuged at 100,000 *g*/1 h/4 °C: pellets were saved as ER proteins and the supernatants as cytosolic ones.

### Mass spectrometry-based quantitative proteomic analysis

Proteins were stacked in a single band in the top of an SDS-PAGE gel (4–12% NuPAGE, Life Technologies) and stained with Coomassie blue R-250 before in-gel digestion using modified trypsin (Promega, sequencing grade) as previously described [52]. Resulting peptides were analyzed by online nanoliquid chromatography coupled to tandem MS (UltiMate 3000 and LTQ-Orbitrap Velos Pro, Thermo Scientific). Peptides were sampled on a 300 µm × 5 mm PepMap C18 precolumn (Thermo Scientific) and separated on a 75 µm × 250 mm C18 column (Reprosil-Pur 120 C18-AQ, 3 μm, Dr. Maisch) using a 120 min gradient. MS and MS/MS data were acquired using Xcalibur (Thermo Scientific). Peptides and proteins were identified and quantified using Mascot (version 2.6.0) using the Uniprot database (Mus musculus taxonomy) and the frequently observed contaminant database embedded in MaxQuant. Trypsin was chosen as the enzyme and two missed cleavages were allowed. Peptide modifications allowed during the search were: carbamidomethylation (C, fixed), acetyl (Protein N-ter, variable), and oxidation (M, variable). The Proline software [6] was used to filter the results: conservation of rank 1 peptides, peptide score ≥ 25, peptide length ≥ 6, peptide-spectrum-match identification false discovery rate < 1% as calculated on scores by employing the reverse database strategy, and minimum of 1 specific peptide per identified protein group. Proline was then used to perform a compilation, grouping, and MS1 quantification of the protein groups using specific peptides. Statistical analysis was performed using ProStaR [68]. Proteins identified in the reverse and contaminant databases, proteins only identified by site, proteins identified by MS/MS in less than two replicates in one condition, and proteins exhibiting less than four intensity values in one condition were discarded from the list. After log2 transformation, abundance values were normalized using the variance stabilizing normalization procedure before missing value imputation (slsa method for partially observed values and DetQuantile with quantile and factor set to 1 for values missing in the entire condition); statistical testing was conducted using limma test. Differentially expressed proteins were sorted out using a log2 (fold change) cut-off of 0.8 and a p value cut-off inferior to 0.05 (allowing to reach a Benjamini–Hochberg FDR inferior to 5%). The mass spectrometry proteomics data have been deposited to the ProteomeXchange Consortium via the PRIDE partner repository [45] with the dataset identifier PXD015280.

Biological processes were determined with the Panther software (pantherdb.org).

### Intramyocardial adenovirus injection

The 4mtD3cpv plasmid was obtained from Dr Roger Tsien’s lab [44], while the D4ER plasmid was a gift from Pr Tullio Pozzan [19] and the reticulum–mitochondria RFP synthetic linker from Dr Gyorgy Hajnoczky [9]. Recombinant adenoviruses were generated by homologous recombination in the VmAdcDNA3 plasmid, amplified and purified, as previously described [11]. One week before cardiomyocyte isolation for Ca^2+^ imaging experiments, mice were anesthetized with 2% isoflurane and supplemented subcutaneously with buprenorphine (0.075 mg/kg). A left thoracotomy was performed in the fourth left intercostal space and the pericardium was opened to expose the heart. 5 × 10^8^ PFU of adenovirus in 20 µl NaCl was injected into the left ventricular wall at seven different points. The chest cavity was closed and the mice were allowed to recover for 1 week prior to cardiomyocyte isolation.

### Immunoprecipitation and western blot

Proteins were extracted from cardiomyocytes or frozen hearts using complete RIPA lysis buffer (150 mM NaCl, 1% Triton X-100, 0.5% sodium deoxycholate, 0.1% SDS, 50 mM Tris-Base, 1 mM DTT, and 5 mM EDTA) and supplemented with anti-protease (Sigma P8340) and anti-phosphatase (Sigma P5726) cocktails. Proteins were quantified with the Lowry DC Protein Assay (Bio-Rad). For IP3R immunoprecipitation, as previously described [43], 500 µg were incubated with 2 µg of IP3R1 antibody (Santa Cruz, sc28614) overnight at 4 °C with rotation in buffer containing 50 mM Tris, 150 mM NaCl, 1% Triton X-100, and 1 mM EDTA supplemented with phosphatase and protease inhibitors (Sigma). The next day, samples were incubated for 2 h with Pure Proteome protein G magnetic beads (Millipore) at 4 °C, and elution was done using 2X Laemmli buffer. SDS-PAGE Western blotting was then performed and membranes were cut into strips to detect several proteins (IP3R1, VDAC, and Grp75) at the same time.

Phospho-AKT, AKT, CHOP, and Grp78 proteins were detected in total heart homogenates (50 µg), phospho-PDH, and PDH proteins were assessed in cardiomyocyte lysates (30 µg), while MICU1 and MCU proteins were detected in pure mitochondria samples (15 µg). Mfn2 and VDAC assessments were done on isolated cardiac MAM samples (15 µg). After sample loading, proteins were transferred onto a nitrocellulose membrane, which was blocked for 1 h with 5% bovine serum albumin (BSA)-PBS. Immunoblotting was performed by incubating the membranes overnight at 4 °C with primary antibodies: rabbit anti-IP3R1 (1/1000; sc28614), mouse anti-VDAC (1/1000; Abcam, ab14734), mouse anti-Grp75 (1/1000; sc133137), rabbit anti-phospho-AKT (1/1000; Cell Signaling 4060L), rabbit anti-AKT (1/1000; Cell Signaling 4691S), mouse anti-CHOP (1/1000; Cell Signaling 2895S), goat anti-Grp78 (1/500; sc1050), rabbit anti-MICU1 (1/500; Sigma HPA037480), rabbit anti-MCU (1/1000; Sigma HPA016480), rabbit anti-MFN2 (1/1000; Abcam ab50838), mouse anti-phospho-PDH (1/2000; Cell Signaling 31866S), and rabbit anti-PDH (1/000; Cell Signaling C54G1) prepared in 5% BSA-PBS. Horseradish peroxidase-conjugated anti-mouse and anti-rabbit secondary antibodies (1/10,000; Biorad) were added for 1 h incubation at RT. Immunoblot development was done using the reaction substrate ECL prime reagent (GE healthcare) with Bio-Rad Molecular Imager Gel Doc XR + (Bio-Rad). Image Lab software (Bio-Rad) was used for quantification.

### Wide-field Ca^2+^ imaging

Mitochondrial and reticular Ca^2+^ concentrations were, respectively, followed by FRET-based Ca^2+^ sensors, 4mtD3cpv and D4ER, expressed in adult mouse cardiomyocytes by in vivo adenoviral delivery and monitored with excitation at 430 nm (CFP) and emission at 485 nm (CFP) and 540 nm (YFP). Live measurements were performed at 37 °C in 1 ml CCB (0.14 M NaCl, 5 mM KCl, 1 mM MgCl_2_, 10 mM HEPES, and 2 mM CaCl_2_ with 10 mM glucose) or 1 ml CFB (similar to CCB without 2 mM CaCl_2_ for D4ER measurements) using a wide-field Leica DMI6000B microscope equipped with a 40 × objective and an Orca-Flash4.0 digital camera (HAMAMATSU). Following stabilization of the resting Ca^2+^ level (from 1 to 2 min), mitochondrial Ca^2+^ uptake following IP3R Ca^2+^ release was induced by 10 µM IP_3_-AM (SiChem). Mitochondrial Ca^2+^ concentration was recorded similarly in a field stimulation buffer (150 mM NaCl, 5.4 mM KCl, 10 mM Hepes, 2 mM MgCl_2_ Anhydrous, 1 mM Glucose, 2.5 mM Pyruvate, 5 mM Creatine, 5 mM Taurine, and 2 mM CaCl_2_) under successive electrical stimulations at 0.5, 1 Hz, and 2 Hz for 1 min each, using the MyoPacer Field Stimulator (IonOptix). Reticular Ca^2+^ release through the ryanodine receptors was induced by 5 mM caffeine (Sigma C0750). YFP/CFP fluorescence ratio was calculated with MetaFluor 6.3 (Universal imaging) after removing background fluorescence. For resting cytosolic Ca^2+^ level measurement, cardiomyocytes were loaded with 5 µM Fura2-AM (Thermo Fisher F1221) in CCB for 30 min at 37 °C, and then washed with CCB, and fluorescence was recorded using 340/380 nm excitation and 510 nm emission.

### Metabolic assays

NADH autofluorescence was recorded at 25 °C in freshly isolated cardiomyocytes (1 mg) in CCB supplemented with 2 mM pyruvate, using a Hitachi F2500 spectrofluorometer with excitation at 360 nm and emission at 450 nm. NADH calibration was achieved by 2 µM rotenone and then 10 µM FCCP for each run.

Oxygen consumption rates (OCR) were monitored in freshly isolated cardiomyocytes (500 µg), in CCB supplemented with 2 mM pyruvate, using an Oroboros respirometer at 25 °C. Maximal respiration level was induced by 10 µM FCCP.

Total ATP content was detected using an ATP Bioluminescence Assay Kit (Roche 11,699,709,001) on freshly isolated adult cardiomyocytes at 16 weeks of diet.

### Field-stimulated cytosolic Ca^2+^ transients and cell shortening

Cytosolic Ca^2+^ transients were recorded measuring the Fluo-4 signal (Thermo Fisher F14201: ex/em 490/510) with a laser scanning confocal microscope (Nikon A1R) on a 40 × objective at 37 °C. Cardiomyocytes loaded with 1 µM Fluo-4 for 30 min at 37 °C, then washed with CCB, were placed in the field stimulation buffer, and electrically stimulated with the MyoPacer Field Stimulator (IonOptix) successively at 0.5 and 1 Hz for 1 min each with a rest of 1 min in between (biphasic pulse, 40 V amplitude, and 0.5 ms delay). Images were acquired every 68 ms, and then, normalization was done to the average resting fluorescence intensity (*F*/*F*_0_). Ca^2+^ transients were analyzed using a homemade program developed with Matlab and Statistics toolbox (version R2014B, The MathWorks Inc.). Transient peaks and minima were detected with a standard algorithm for finding local extrema based on the derivative of the signal and the corresponding amplitude and time of each extremum were recorded. From these values, different parameters were measured: time to peak, half-time, and time peak to basal. Cardiomyocyte shortening was evaluated in Fluo-4 loaded cells by line scanning along the long axis. Cell length was measured, using ImageJ software, in the resting state and in the maximally contracted state to express the shortening as a percentage of the resting cell length.

### Echocardiography

Echocardiography was performed under a light anesthesia (ketamine 80 mg/kg ip), with a digital ultrasound system (Vivid 7, GE Medical Systems) and a 13-MHz linear-array transducer as previously described [61]. LV end-diastolic diameter (LVEDD), LV end-systolic diameter (LVESD), fractional shortening (FS), anterior wall thickness (AWT), and posterior wall thickness (PWT) were obtained from M-mode tracings at the level of the papillary muscles. LV mass was calculated using the following formula: 1.05 × [(AWT + PWT + LVEDD)3 − LVEDD3] [37, 48]. Strain-rate images were obtained from the parasternal short-axis views at the midventricular level as previously described [56, 60]. Echography analyses were performed offline by an observer blinded to the groups with the use of the EchoPac Software (GE Medical).

For diastolic function, mice were anesthetized with 2–3% isoflurane to set the heart rate between 360 and 450 bpm (SD: 414 ± 14 bpm vs HFHSD: 428 ± 39 bpm, Mean ± SEM, *N* = 9, *p* = ns). Acquisitions were performed on the Vevo 3100 imaging system using a 40-MHz linear probe (VisualSonics). E and A peak velocity was measured from the mitral flow profiles obtained by Pulsed Wave Doppler in the apical four-chamber view as well as isovolumic relaxation time (IVRT), while Tissue Doppler imaging at the septal corner of the mitral valve annulus was performed to assess E’ peak velocity. Analyses were performed offline blindly on the VevoLab software, as previously described [55].

### Statistical analysis

Parametric t test and one-way ANOVA were performed for statistical analysis of echocardiography measurements (from the table), after validation of the Shapiro–Wilk normality test: these data are presented as mean ± SEM. For all the other analyses, non-parametric tests were used: Mann–Whitney test for two groups, Kruskal–Wallis test followed by the Dunn’s multiple comparisons test for three groups as a one-way ANOVA with only one variable, whereas for two variables, a two-way ANOVA followed by a Sidak or a Tukey’s post hoc test were applied. Data are thus expressed as median (interquartile range: P[25], P[75]). Experiments were performed at least in three different mice per group (= N), with the two (SD, HFHSD) or three groups (SD, HFHSD, HFHSD/SD) each day. For single-cell imaging analysis, statistics were performed on *n* = number of cells to assess single-cell effect as well as heterogeneity between them. Both *N* and *n* values are indicated in the figure legends. A *p* value < 0.05 was considered significant.

## Results

### 16 weeks of HFHSD led to a cardiac insulin resistance and to an early phenotype of diabetic cardiomyopathy

**Fig. 1:**
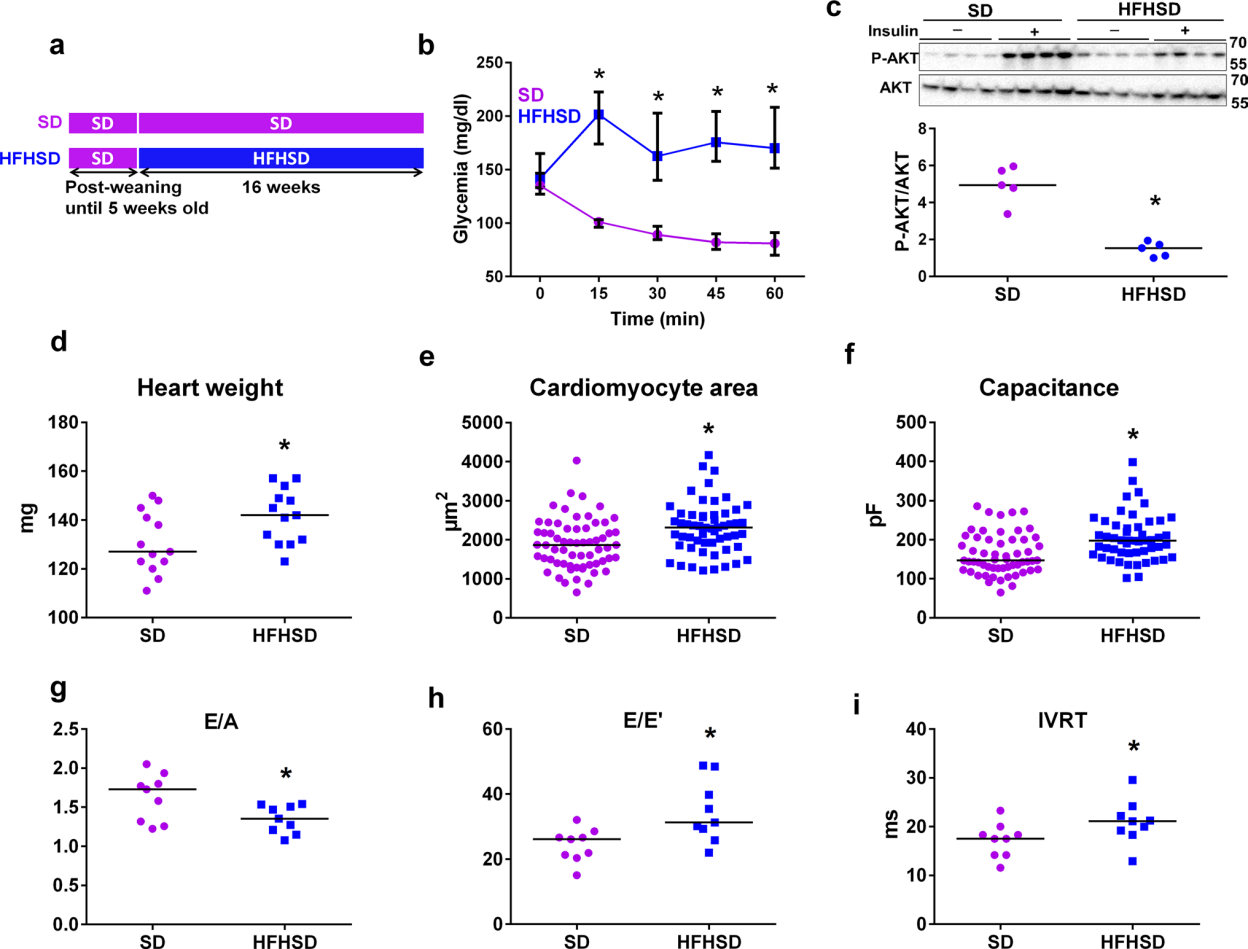
Diet-induced T2D altered the cardiac insulin signaling and triggered an early diabetic cardiomyopathy phenotype. **a** Study design of the diet groups: at 5 weeks old, mice were assigned to 16 weeks of either Standard diet (SD) or High-Fat High-Sucrose diet (HFHSD). **b** Insulin tolerance assessment by following glycemia levels after an intraperitoneal injection of insulin (0.75 mU/g) (*N* = 10 mice per group). **c** Cardiac pAKT/AKT levels reflecting the cardiac insulin sensitivity. Upper panel: representative immunoblots. Lower panel: quantification of P-AKT/AKT as a fold of insulin-induced AKT phosphorylation over NaCl (*N* = 5 mice per group). **d** Measurement of heart weight after 16 weeks of SD or HFHSD (*N* = 13 mice/group; Mann–Whitney test). **e** Quantification of SD and HFHSD cardiomyocyte area using ImageJ software (*n* = 56–63 cells from *N* = 4 mice/group, Mann–Whitney test; *p* < 0.05 when statistics made on *N* = 4 mice/group). **f** Cardiomyocyte membrane capacitance recorded by electrophysiology patch clamp (*n* = 51–57 cells from *N* = 5 mice/group, Mann–Whitney test; *p* < 0.05 when statistics made on *N* = 5 mice/group). **g to i** Echocardiography assessment of diastolic function: quantification of E/A ratio **(g)**, E/E’ ratio **(h)** and isovolumic relaxation time, IVRT **(i)** from *N* = 9 mice/group. Data are presented as median; * *p* < 0.05 versus SD

To assess the effect of obesity and T2D on cardiac insulin signaling, we used the diet-induced obesogenic T2D mouse model with animals receiving a high-fat high-sucrose diet (HFHSD) for 16 weeks (Fig. 1a), as previously validated by our group [65, 66]. Following 16 weeks of such a diet, the HFHSD mice displayed an increased body weight (Table 1), and were both hyperinsulinemic and hyperglycemic (SD: 0.55 [0.46, 2.50] versus HFHSD: 3.72 [2.71, 3.78] ng insulin / ml plasma; SD: 89 [72, 103] versus HFHSD: 115 [101, 130] mg glucose / dl blood, *N* = 8 mice/group). Systemic insulin and glucose resistance was shown by the absence of glycemic regulation during the insulin and glucose tolerance tests (Fig. 1b and Supplementary Fig. 1a) when compared to the SD-fed mice. Importantly, 16 weeks of HFHSD led to a decreased insulin-stimulated Ser473-AKT phosphorylation in the heart (Fig. 1c), reflecting a cardiac insulin resistance.

**Table 1:** Echocardiography analysis of SD, HFHSD, and HFHSD/SD mice at 16 and 24 weeks of diet

	16 weeks		24 weeks		
	SD	HFHSD	SD	HFHSD	HFHSD/SD
Body weight, g	29.13 ± 0.56	42.91 ± 0.89	31.57 ± 0.75	47.63 ± 0.74	36.52 ± 0.88
Heart rate, bpm	588 ± 11	621 ± 15	609 ± 18	634 ± 15	641 ± 10
LV mass, mg	91 ± 3	103 ± 4*	82 ± 3	115 ± 4*	98 ± 4*†
AWD thickness, mm	0.79 ± 0.01	0.80 ± 0.02	0.81 ± 0.02	1.01 ± 0.03*	0.85 ± 0.02†
LVEDD, mm	3.59 ± 0.07	3.60 ± 0.05	3.31 ± 0.06	3.24 ± 0.06	3.37 ± 0.03
LVESD, mm	1.70 ± 0.06	1.60 ± 0.09	1.46 ± 0.14	1.51 ± 0.07	1.36 ± 0.07
PWD thickness, mm	0.77 ± 0.01	0.90 ± 0.02*	0.79 ± 0.02	1.041 ± 0.03*	0.92 ± 0.03†
LV FS, %	52.60 ± 1.22	55.70 ± 2.31	55.83 ± 4.03	53.33 ± 1.89	59.80 ± 2.02
PWSR, unit/s	22.38 ± 0.86	21.06 ± 1.01	27.17 ± 1.60	21.80 ± 1.68	30.50 ± 1.37†
AWSR, unit/s	20.50 ± 1.47	17.40 ± 1.12	24.17 ± 1.25	15.87 ± 1.66*	23.10 ± 2.34†

Heart rate, left-ventricular (LV) mass, anterior wall diastolic (AWD) thickness, left-ventricular dimension [end-diastolic diameter (LVEDD) and end-systolic diameter (LVESD)], left-ventricular posterior wall diastolic thickness (PWD), fractional shortening (FS), and posterior and anterior walls strain rates (PWSR and AWSR, respectively) were measured by echocardiography in SD and HFHSD mice at 16 weeks (*N* = 10), as well as 8 weeks later for SD (*N* = 6), HFHSD (*N* = 15) and HFHSD/SD (*N* = 10) mice

Data are presented as mean ± SEM; *p < 0.05 vs corresponding SD; †*p* < 0.05 vs corresponding HFHSD

Early features of diabetic cardiomyopathy include hypertrophy and fibrosis [24, 25]. Following 16 weeks of HFHSD, mice displayed a significantly increased heart weight (Fig. 1d), an increased cardiomyocyte area (Fig. 1e), and a higher membrane capacitance (Fig. 1f) when compared to SD mice. Echocardiography showed a thicker posterior wall and a greater left-ventricular mass in HFHSD versus SD mice (Table 1). Of note, HFHSD was not associated with any significant increase in blood pressure (98.1 [89.0, 105.7] in SD versus 96.9 [94.0, 119.8] mmHg in HFHSD, *N* = 8 mice/group, *p* = ns). Masson’s trichrome staining revealed a significant increase in tissue fibrosis in HFHSD versus SD hearts (Supplementary Fig. 1b–c), with an increase in cardiac triglyceride concentration (SD: 10.5 [8.4, 10.7] vs HFHSD: 17.4 [14.5, 23.7] µM/mg of tissue, *N* = 5 mice/group, p < 0.05).

At 16 weeks, HFHSD mice presented a significant diastolic dysfunction, evidenced by a decreased E/A ratio, and increased E/E’ ratio and isovolumic relaxation time (Fig. 1g-i, Supplementary Fig. 1d). They also exhibited a trend towards a decreased strain rate in both anterior and posterior walls as compared to SD mice. This difference in regional wall function became significant at 24 weeks of diet (Table 1). Our results suggest that 16 weeks of HFHSD induced a cardiac fibrosis, lipid accumulation, and heart hypertrophy, together with a diastolic dysfunction and a mild systolic dysfunction.

### Diet-induced obesity and T2D alter cardiac MAM thickness and composition

**Fig. 2:**
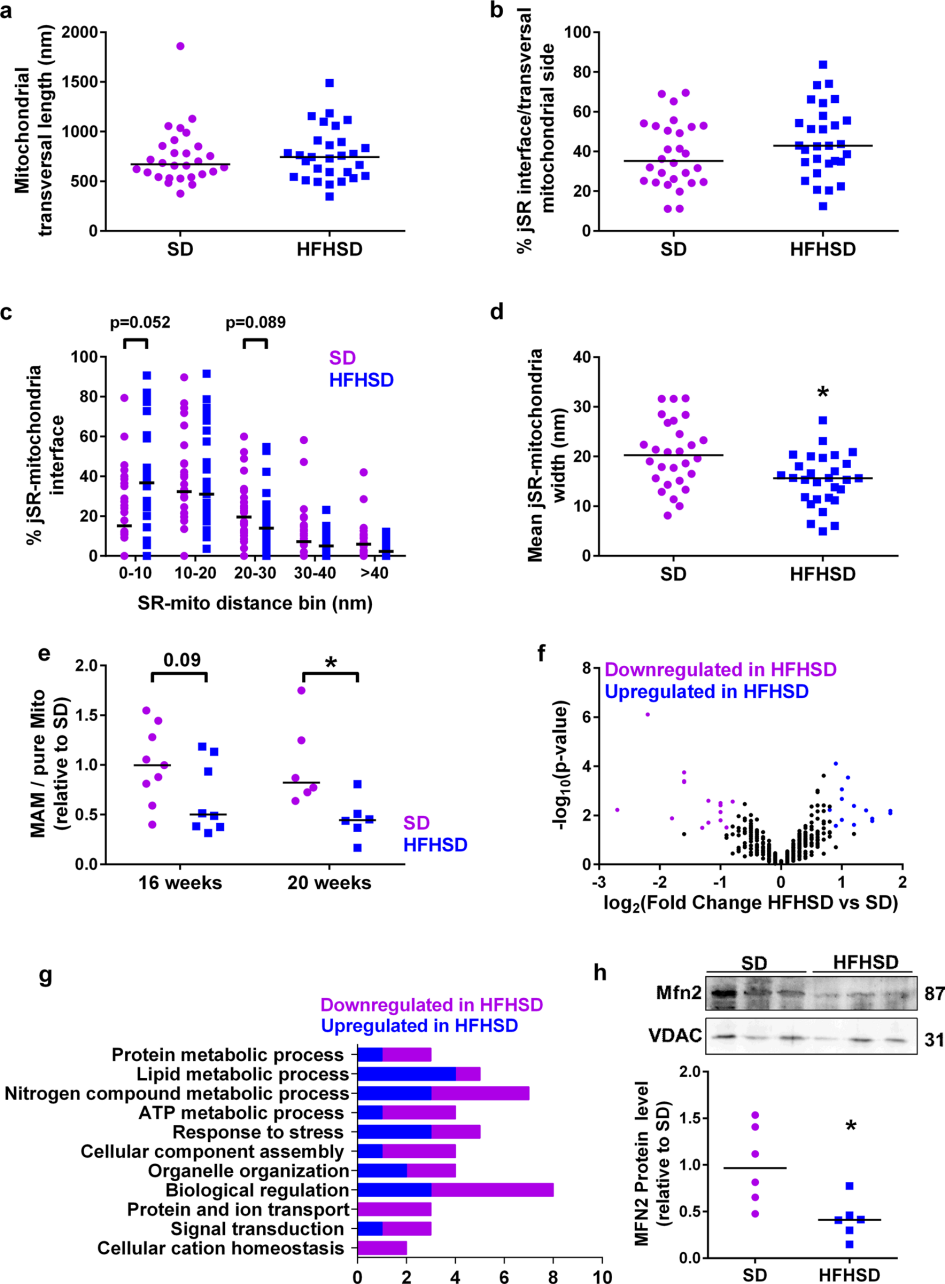
16 weeks of HFHSD altered the reticulum–mitochondria coupling and protein composition. **a-d** Ultrastructural analysis by electron microscopy of the cardiac junctional reticulum (jSR)–mitochondria interactions in SD and HFHSD cardiomyocytes (n = 29 contacts from *N* = 4 mice per group at 16 weeks of diet). Quantification of the length of the mitochondrial transversal side **(a)** and of the jSR–mitochondria interface **(b)**. **c** Bar graph shows the frequency of jSR–mitochondria interaction width. **e** Quantification of the SD-relative protein ratio of MAM over pure mitochondria from heart fractionation at 16 and 20 weeks of diet (*N* = 8–9 mice per group at 16 weeks; *N* = 6 mice per group at 20 weeks). **f** Volcano plot of the 522 proteins identified in the SD and HFHSD MAM proteome (*N* = 4 mice per group at 16 weeks). Each point represents an individual protein. Significant upregulated and downregulated proteins in HFHSD versus SD are depicted, respectively, in blue and purple. **g** Bar graph showing the functional annotation of the major processes differentially expressed in SD and HFHSD cardiac MAM. **h** Quantification of MFN2 immunoblotting in cardiac MAM isolated from SD and HFHSD mice at 16 weeks of diet (*N* = 6 mice per group). Normalization was done to VDAC. Results are presented as median; Mann–Whitney non-parametric test was performed; *p < 0.05 versus corresponding SD

To determine a putative effect of cardiac insulin resistance on heart MAM coupling, associations between junctional reticulum (jSR) and mitochondria at the ultrastructural level were determined in electron micrographs of SD and HFHSD cardiomyocytes at 16 weeks (Supplementary Fig. 2a–b). Length of the mitochondrial transversal side and of the jSR–mitochondria interface was similar in both groups (Fig. 2a–b). However, a comparison of the occurrence of interactions within a given gap width, ranging from 0 to 100 nm, revealed an increase in tight junctions (0–10 nm) and reduced 20–30-nm MAM interactions in the HFHSD cardiomyocytes (Fig. 2c). Tighter MAM interactions in the HFHSD cardiac cells were further supported by the significantly decreased mean jSR–mitochondria interface width distance (Fig. 2d). Interestingly, specific functions at specific width of the MAM interface were reported, notably lipid metabolism under 10 nm, while Ca^2+^ transfer occurs mainly in the 20–30 nm range [17]. Therefore, we performed an enriched MAM isolation by heart fractionation, whose purity was previously confirmed [43], to analyze the cardiac MAM proteome by MS-based quantitative proteomics. Quantification of the protein content revealed a decrease in the ratio of total MAM over pure mitochondria amount in the HFHSD mouse hearts compared to the SD hearts. This decrease was detectable as soon as after 16 weeks of diet and averaged 50% of SD value at 20 weeks (Fig. 2e). Importantly, no significant change in the total pure mitochondrial content was observed between the two groups whatever the diet duration (Supplementary Fig. 2c). The Volcano plot of the cardiac MAM proteome visually highlights that 14 proteins were significantly ≥ 1.75 times upregulated and 15 proteins ≥ 1.75 times downregulated compared to the SD group (Fig. 2f, Supplementary Table 1). Functional annotation of MAM proteins was performed using the Panther software, revealing an upregulation of lipid metabolic and response to stress processes in the HFHSD model compared to SD (Fig. 2g, Supplementary Table 2). On the other hand, ATP metabolic process, protein/ion transport, and cation homeostasis were downregulated in the HFHSD cardiac MAM. A significant decrease in the protein expression of the known MAM-tethering protein MFN2 was also observed in the HFHSD cardiac MAM (Fig. 2h). However, proteomic analysis revealed no significant changes in VDAC1, Grp75, RyR2, and Serca2 expression in the HFHSD MAM (Supplementary Table 1). Altogether, these data suggest a tighter thickness of the HFHSD cardiac MAM, leading to protein composition alterations notably at the level of Ca^2+^ transport process.

### T2D reduces IP3-stimulated Ca^2+^ transfer to mitochondria and alters mitochondrial bioenergetics in cardiomyocytes

**Fig. 3:**
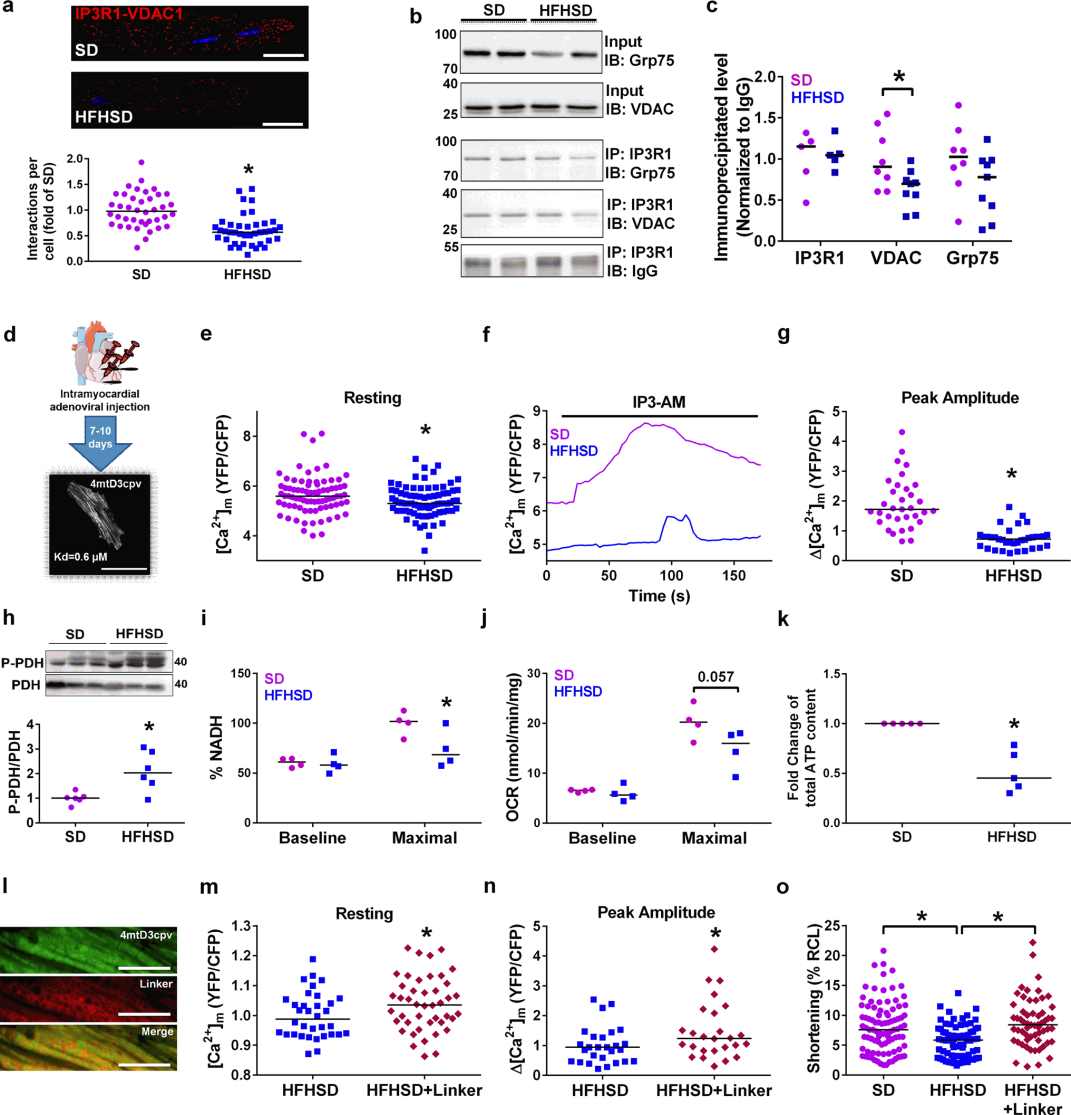
Decreased formation of the IP3R1/Grp75/VDAC Ca^2+^ channeling complex leading to reduced IP3-stimulated Ca^2+^ transfer to mitochondria in the diabetic cardiomyocyte**. a** Proximity ligation assay between IP3R1 and VDAC1 in isolated SD and HFHSD cardiomyocytes. Upper panel: representative confocal microscopy images of in situ IP3R1–VDAC1 interactions depicted as red dots. Nuclei appear in blue. Scale bar: 25 µm. Lower panel: quantification of the interactions per cell presented as a fold of SD (*n* = 40 cardiomyocytes from *N* = 4 mice/group at 16 weeks of diet, Mann–Whitney test; *p *< 0.05 when statistics made on *N* = 4 mice/group). **b** Representative immunoblots of VDAC and Grp75 following IP3R immunoprecipitation in SD and HFHSD cardiomyocytes. **c** Densitometric analysis of IP3R1, Grp75, and VDAC protein levels pulled down by IP3R and normalized to IgG (*N* = 5–8 mice/group at 16 weeks of diet; Mann–Whitney test). **d** Illustration of the in vivo adenoviral strategy to express the mitochondrial Ca^2+^ sensor, 4mtD3cpv, in adult mouse cardiomyocytes. Ten days following direct intramyocardial injection of the adenovirus, isolated cardiomyocytes present a typical mitochondrial pattern of the fluorescent sensor as displayed on the representative confocal image. Scale bar: 50 µm. **e** Quantification of the resting [Ca^2+^]_m_ fluorescence ratio (*n* = 78–81 cells from *N* = 8 mice/group at 16 weeks of diet; Mann–Whitney test;* p* < 0.05 when statistics made on *N* = 8 mice/group). **f** Representative traces of the [Ca^2+^]_m_ as a YFP/CFP ratio, after IP3R stimulation by 10 µM IP3-AM in SD and HFHD cardiomyocytes. **g** Calculations of the [Ca^2+^]_m_ IP3-induced peak amplitude from data in **(f)** (*n* = 33–35 cells from *N* = 4 mice/group at 16 weeks of diet; Mann–Whitney test; *p* < 0.05 when statistics made on *N* = 4 mice/group). Peak amplitude was defined by subtracting the resting [Ca^2+^]_m_ to the [Ca^2+^]_m_ peak level. **h** Quantification of the phosphorylation of PDH over total PDH, relative to SD, in total cardiomyocytes lysates (N = 6 mice per group at 16 weeks of diet; Mann–Whitney test). **i** NADH autofluorescence measurements in intact cardiomyocytes. Baseline NADH autofluorescence level, expressed as a percentage by calibration with rotenone (maximal) and FCCP (minimal). Maximal NADH autofluorescence level after rotenone treatment, calculated as a percentage of increase between baseline and post-rotenone, normalized to SD (*N* = 4 mice per group at 16 weeks of diet; Mann–Whitney test). **j** OCR using the Oroboros respirometer: baseline and FCCP-induced maximal oxidative phosphorylation were measured in intact cardiomyocytes (N = 4 mice per group at 16 weeks of diet; Mann–Whitney test). **k** Fold change of total ATP content in freshly isolated SD and HFHSD cardiomyocytes. HFHSD values are normalized to the respective SD values of the experimental day (*N* = 5 mice per group at 16 weeks of diet; Mann–Whitney test). **l** Representative confocal images of co-infection with the linker construct and the mitochondrial Ca^2+^ sensor 4mtD3cpv. Scale bar: 10 µm. **m** and **n** Quantification of the resting [Ca^2+^]_m_ fluorescence ratio and [Ca^2+^]_m_ IP3-induced peak amplitude, respectively (*n* = 34–44 cells for baseline and n = 26 cells for peak amplitude from *N* = 3–4 mice/group at 16 weeks of diet, Mann–Whitney test; *p* = ns when statistics made on *N* = 3–4 mice/group). **o** Cardiomyocyte contractility under 1 Hz field stimulation, presented as a percentage of the resting cell length (RCL) (*n* = 59–91 cells from *N* = 3–4 mice/group at 16 weeks of diet, Kruskal–Wallis test; *p* = ns when statistics made on *N* = 3–4 mice/group). Data are shown as median; *p < 0.05

Given the reported role of the reticulum–mitochondria interface as a hotspot for cell functions, notably for Ca^2+^ signaling through the IP3R1/Grp75/VDAC1 complex [58], we hypothesized that the HFHSD might alter the IP3R1 Ca^2+^ channeling complex. Proximity ligation assay (PLA) between IP3R1 and VDAC1 in isolated adult cardiomyocytes revealed a decreased number of in situ protein–protein interactions in HFHSD versus SD mice (Fig. 3a). IP3R1 immunoprecipitation further confirmed a reduced pull-down of both VDAC1 and Grp75 in the HFHSD heart (Fig. 3b-c), while total levels of these proteins were not changed (Supplementary Fig. 3a). These results suggest a decreased formation of the IP3R1/Grp75/VDAC Ca^2+^ channeling complex in the HFHSD cardiomyocyte.

We next investigated the functional consequences of this structural modification. Since adult mouse cardiomyocytes cannot be cultured for more than 1 day without ultrastructural changes (notably mitochondrial) [14, 32], we developed an in vivo adenoviral delivery strategy, relying on the intramyocardial injection of an adenovirus encoding a FRET-based mitochondrial Ca^2+^ sensor, 4mtD3cpv (Fig. 3d). One week after the injection, isolated HFHSD cardiomyocytes displayed a 5.3% lower resting mitochondrial Ca^2+^ concentration ([Ca^2+^]_m_) compared to SD cells (Fig. 3e). IP3R-mediated Ca^2+^ transfer into mitochondria was then measured following 10 µM IP3-AM stimulation (Fig. 3f). A significant 58% reduction of the [Ca^2+^]_m_ peak amplitude was observed in the HFHSD versus SD cardiomyocytes (Fig. 3g).

Since mitochondrial Ca^2+^ is a key regulator of mitochondrial energetics and notably mitochondrial ATP production, we analyzed the phosphorylation status of the pyruvate dehydrogenase (PDH) and observed a significant increase in PDH phosphorylation in the HFHSD cardiomyocytes (Fig. 3h), suggesting an altered mitochondrial energy production. Monitoring of NADH autofluorescence showed no change at baseline between SD and HFHSD cardiomyocytes, but maximal NADH production elicited by rotenone was decreased in the HFHSD cells (Fig. 3i). To evaluate the impact of HFHSD on the oxidative phosphorylation and thus ATP production, oxygen consumption rates (OCR) were measured in intact cardiomyocytes. While no change in baseline respiration was observed, FCCP-induced maximal OCR were reduced in the HFHSD cardiomyocytes (Fig. 3j). It was associated with a significant decrease in total ATP content in the HFHSD versus SD freshly isolated cardiomyocytes (Fig. 3k). Interestingly, measurement of OCR in digitonin-permeabilized cardiomyocytes, which allows a control of the medium surrounding the mitochondria with a partial loss of reticulum contribution, did not show any changes in either state 2 or state 3 respiratory rates (Supplementary Fig. 3b–c). Altogether, these data support decreased mitochondrial bioenergetics in the HFHSD cardiomyocytes without alteration of the electron transport chain but rather due to the deficient reticulum–mitochondria Ca^2+^ transfer.

However, while superoxide ion production was slightly increased in HFHSD cardiomyocytes, no change was observed at the level of carbonylated cardiomyocyte proteins (Supplementary Fig. 3d–f), indicating a mild oxidative stress at this stage. In summary, our data validate that the T2D-induced cardiac MAM thickness alteration results in a decreased IP3-stimulated Ca^2+^ transfer to heart mitochondria due to a reduced formation of the IP3R1/Grp75/VDAC1 Ca^2+^ channeling complex, leading to the disruption of mitochondrial bioenergetics, yet not to an oxidative stress exacerbation.

### Reestablishing reticulum–mitochondria interactions improves Ca^2+^ transfer to mitochondria and function of HFHSD cardiomyocytes

In an attempt to demonstrate the cause-and-effect mechanistic link between MAMs functional Ca^2+^ uncoupling and cardiac dysfunction through an altered mitochondrial Ca^2+^ handling, we wondered whether bringing reticulum and mitochondria to the adequate width (20–30 nm range) will rescue the IP3-driven mitochondrial Ca^2+^ uptake and improve HFHSD cardiomyocyte function. To this end, we performed intramyocardial adenoviral injection to co-express the 4mtD3cpv mitochondrial Ca^2+^ sensor with a constitutive synthetic reticulum–mitochondria linker in HFHSD mice (Fig. 3l), as previously validated [1, 9]. MAM linker-expressing HFHSD cardiomyocytes displayed a significantly higher basal and IP3-stimulated [Ca^2+^]_m_ (Fig. 3m-n, respectively 5.1% and 30.8%) compared to mCherry-infected HFHSD cardiomyocytes. Assessment of cardiomyocyte shortening under field stimulation revealed a rescue of cardiomyocyte contractility in the HFHSD + linker group to the level of SD cardiomyocytes (Fig. 3o, 23% decrease of HFHSD vs SD and 43.5% increase in HFHSD + Linker vs HFHSD). These data demonstrate first the reversibility of the diet-induced calcium and contractile diabetic phenotype, and second, the determinant role of the reticulum–mitochondria functional Ca^2+^ miscoupling on mitochondrial Ca^2+^ mishandling and the ensuing cardiomyocyte contractile dysfunction.

### Reduced field-stimulated mitochondrial Ca^2+^ transfer after 16 weeks of HFHSD with maintenance of the reticular Ca^2+^ stock and the cardiac excitation–contraction coupling

**Fig. 4:**
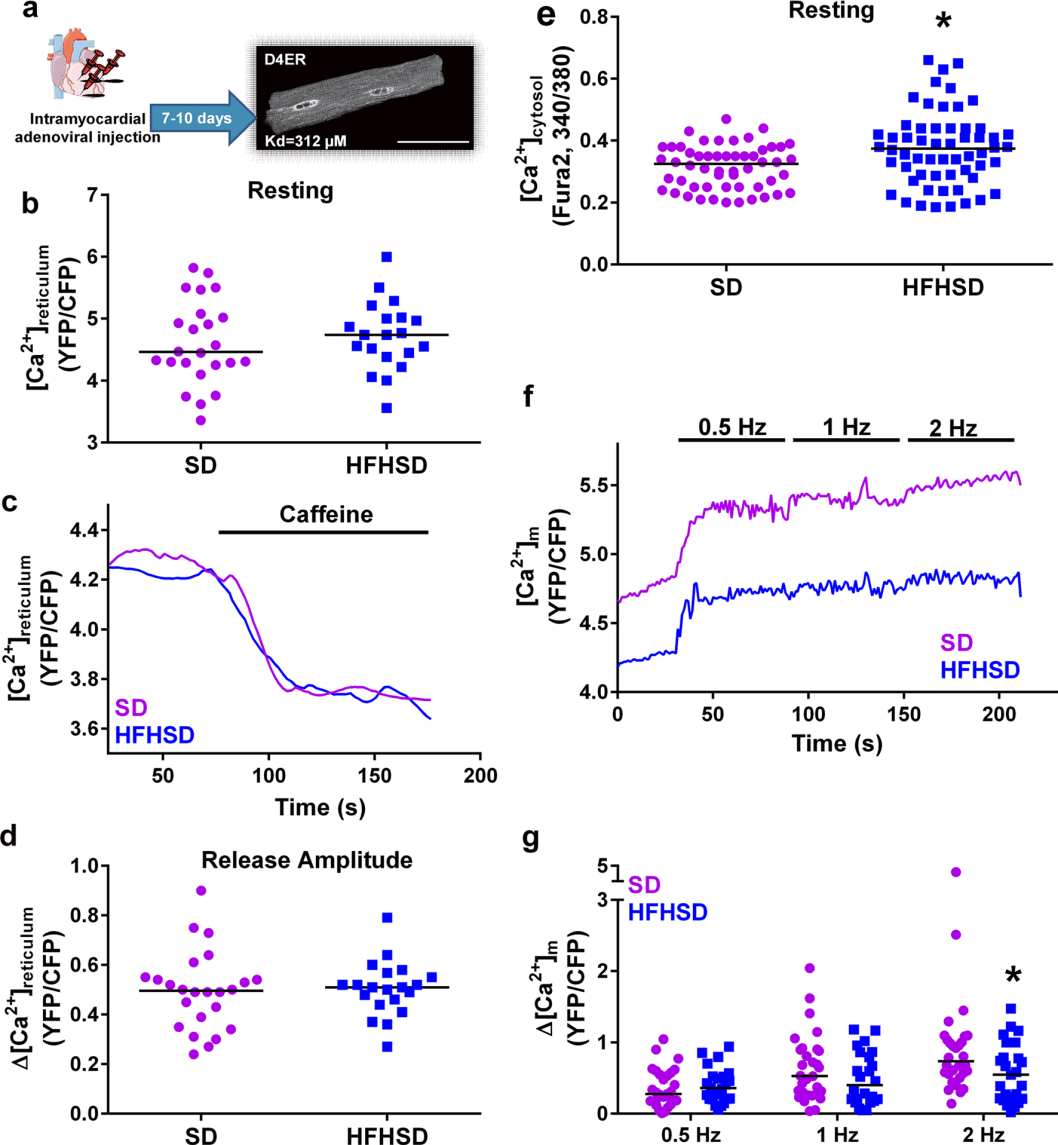
Effect of 16 weeks of HFHSD on the whole cardiomyocyte Ca^2+^ homeostasis. **a** Illustration of the intramyocardial adenoviral injection of the reticular Ca^2+^ sensor D4ER, with a representative confocal image of an infected cardiomyocyte displaying a reticular pattern. Scale bar: 50 µm. **b** Dot plot shows the resting reticular [Ca^2+^] measured in infected cardiomyocytes under resting conditions (*n* = 20–25 cells from *N* = 4 mice/group at 16 weeks of diet, Mann–Whitney test; *p* = ns when statistics made on *N* = 4 mice/group). **c** Representative time course of the reticular [Ca^2+^] after RyR stimulation with 5 mM caffeine in SD and HFHD cardiomyocytes. **d** Summary data show the level of reticular [Ca^2+^] released by caffeine addition in **(c)** (*n* = 20–25 cells from *N* = 4 mice/group at 16 weeks of diet, Mann–Whitney test; *p* = ns when statistics made on *N* = 4 mice/group). **e** Measurement of [Ca^2+^]_c_ levels in Fura2-loaded cardiomyocytes (*n* = 55–58 cells from *N* = 4 mice/group at 16 weeks of diet, Mann–Whitney test; *p* = ns when statistics made on *N* = 4 mice/group.**) f** Representative traces of [Ca^2+^]_m_ after field stimulation with 0.5, 1, and 2 Hz. **g** Calculations of the [Ca^2+^]_m_ field stimulation-induced peak amplitude from data in **(f)** (*n* = 26–34 cells from *N* = 3 mice/group at 16 weeks of diet, Sidak multiple comparison test; *p* = 0.05 at 2 Hz when statistics made on *N* = 3 mice/group). Results are displayed as median;*p < 0.05 versus SD

One could wonder whether a decreased reticular Ca^2+^ stock could also be involved in the reduced IP3-stimulated Ca^2+^ transfer in the HFHSD mouse heart. To this end, we employed intramyocardial adenoviral injection to express a FRET-based reticular Ca^2+^ sensor, D4ER (Fig. 4a). No significant change in the reticular [Ca^2+^] was observed in resting conditions (Fig. 4b). Stimulation by caffeine (5 mM) to empty the reticular Ca^2+^ stock led to a similar Ca^2+^ release in SD and HFHSD cardiomyocytes (Fig. 4c-d), suggesting an absence of alteration of the Ca^2+^ levels in the reticulum. It is, therefore, unlikely that the observed reduction of Ca^2+^ exchange between reticulum and mitochondria was related to a reduction of the reticular Ca^2+^ stock. Measurement of the cytosolic [Ca^2+^] ([Ca^2+^]_c_) using the ratiometric probe Fura2-AM showed a significant small increase of 15.6% in the resting [Ca^2+^]_c_ in HFHSD (Fig. 4e), supporting that reticular Ca^2+^ was directed towards the cytosolic rather than the mitochondrial compartment in HFHSD cardiomyocytes.

To determine if the excitation–contraction coupling was altered following 16-week HFHSD, we performed electrophysiological recordings of inward L-type Ca^2+^ current (I_Ca,L_) in ventricular myocytes from SD and HFHSD cells (Supplementary Fig. 4a). Supplementary Fig. 4b shows the current–voltage relationships of normalized I_Ca,L_ peak to membrane capacitance from SD and HFHSD cells. No significant difference in the density of I_Ca,L_ was observed between the two groups, nor in the density of the Na^+^/Ca^2+^ exchange current measured as the lithium-sensitive slow tail current (Supplementary Fig. 4c–d). Monitoring of [Ca^2+^]_c_ by Fluo4-AM in cardiomyocytes under field stimulation at both 0.5 and 1 Hz did not reveal any significant differences in the cytosolic Ca^2+^ transients, including the peak amplitude, the time to peak, the half-time, and the time peak to basal (Supplementary Table 3). However, the examination of [Ca^2+^]_m_ in paced cardiomyocytes (Fig. 4f) revealed a reduced Ca^2+^ transfer in HFHSD cardiomyocytes, becoming clearly significant at 2 Hz (Fig. 4g), and confirmed by a significant decrease in the area under curve (SD: 67.3 [45.7, 88.7] vs HFHSD: 45.4 [26.8, 58.3] a.u, *n* = 26–34 cells from *N* = 3 mice/group, *p* < 0.05). These results support that the diet-induced functional alterations of MAM coupling precede any dysfunction of the excitation–contraction coupling at both plasma membrane and cytosolic levels while altering the field-stimulated physiological mitochondrial Ca^2+^ transfer.

### Sixteen weeks of HFHSD does not alter the mitochondrial Ca^2+^ uniporter

**Fig. 5:**
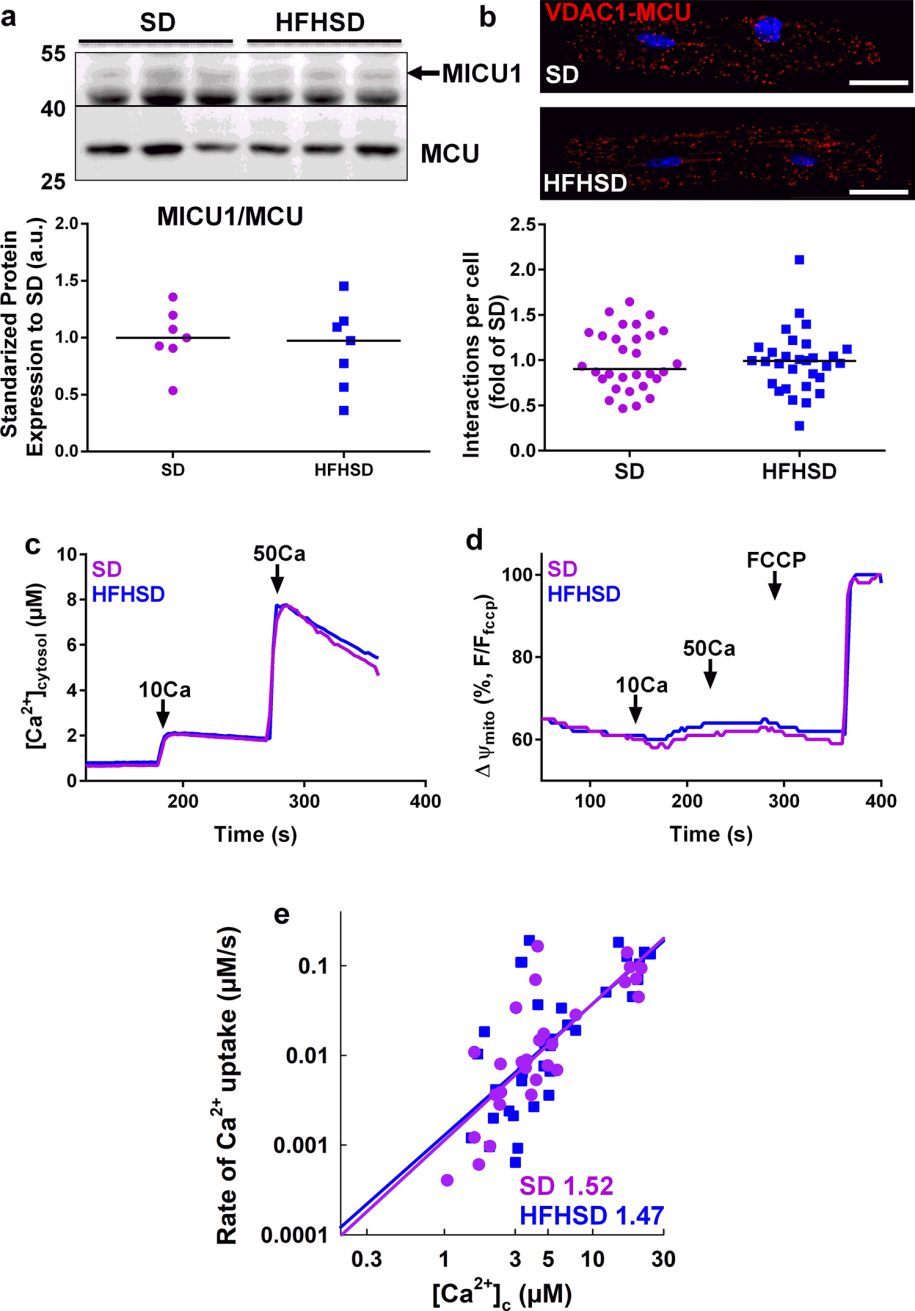
No change in the composition and function of the cardiac mitochondrial Ca^2+^ uniporter after 16 weeks of HFHSD. **a** Detection of MICU1 and MCU by immunoblotting in isolated cardiac pure mitochondria from SD and HFHSD hearts. Lower panel displays the densitometric analysis of the MICU1 to MCU ratio, being normalized to SD (*N* = 7–8 mice per group at 16 weeks of diet; Mann–Whitney test). **b** Representative confocal microscopy images of the VDAC1–MCU interactions in SD and HFHSD cardiomyocytes by proximity ligation assay, scale bar: 25 µm. Quantification of the interactions per cell (fold of SD) is presented as a dot plot (*n* = 40 cells from *N* = 3 mice per group at 16 weeks of diet, Mann–Whitney test; *p* = ns when statistics made on *N* = 3 mice/group). **c** Time courses of the mitochondrial clearance of the [Ca^2+^]_c_ rise, measured by Fura2, upon addition of CaCl_2_ bolus (10 µM followed by 50 µM) in suspensions of SD or HFHSD heart mitochondria. **d** Simultaneous recordings of mitochondrial membrane potential with **(c)**, measured by TMRM and calibrated for maximal depolarization with 2 µM FCCP. **e** Double logarithmic plot of the initial Ca^2+^ uptake rates against the measured peak [Ca^2+^]_c_ in SD and HFHSD cardiac mitochondria (*N* = 4 mice per group at 16 weeks of diet; Mann–Whitney test). Slope of each linear fit is indicated. Results are shown as median.

We questioned whether HFHSD might affect directly the mitochondrial Ca^2+^ uniporter, which is responsible for the Ca^2+^ entry into the mitochondrial matrix. First, we analyzed the protein level of the pore-forming protein MCU and its main regulator MICU1, since the stoichiometry of the MICU1 to MCU ratio directly controls the function of the mitochondrial Ca^2+^ uniporter [42]. Sixteen weeks of HFHSD did not change the MICU1/MCU protein expression measured in isolated cardiac mitochondria (Fig. 5a). In addition, the proximity between VDAC and MCU remained unchanged, as assessed by in situ PLA (Fig. 5b).

We next investigated the direct mitochondrial Ca^2+^ uptake through the mitochondrial Ca^2+^ uniporter. We measured the ruthenium red-sensitive clearance of Ca^2+^ added to the cytoplasmic-like medium in SD and HFHSD isolated heart mitochondria, in the presence of both thapsigargin and CGP37157 to, respectively, inhibit the sarco/endoplasmic reticulum Ca^2+^-ATPase and the mitochondrial Na^+^/Ca^2+^ exchanger, as previously done [42]. When exposed to micromolar [Ca^2+^]_c_, a rapid mitochondrial Ca^2+^ clearance was recorded in both SD and HFHSD cardiac mitochondria (Fig. 5c). Importantly, simultaneous measurements of mitochondrial membrane potential showed similar mitochondrial polarization, indicating that the mitochondrial driving force was not limiting the Ca^2+^ uptake (Fig. 5d). To directly determine the dependence of SD and HFHSD heart mitochondrial uptake on [Ca^2+^]_c_, we constructed a double logarithmic plot of the initial Ca^2+^ uptake rates against varying [Ca^2+^]_c_. We did not observe any change in the threshold or in the cooperative activation, since the slopes of the linear fit were comparable (Fig. 5e; SD: 1.52 vs HFHSD: 1.47). Thus, 16 weeks of HFHSD did not modify the composition and the function of the mitochondrial Ca^2+^ uniporter in the heart, indicating that the decreased mitochondrial Ca^2+^ uptake in HFHSD cardiomyocytes is due mainly to the reduced reticulum–mitochondrial functional Ca^2+^ coupling.

### Diet reversal alleviates the T2D-induced cardiac dysfunction

**Fig. 6:**
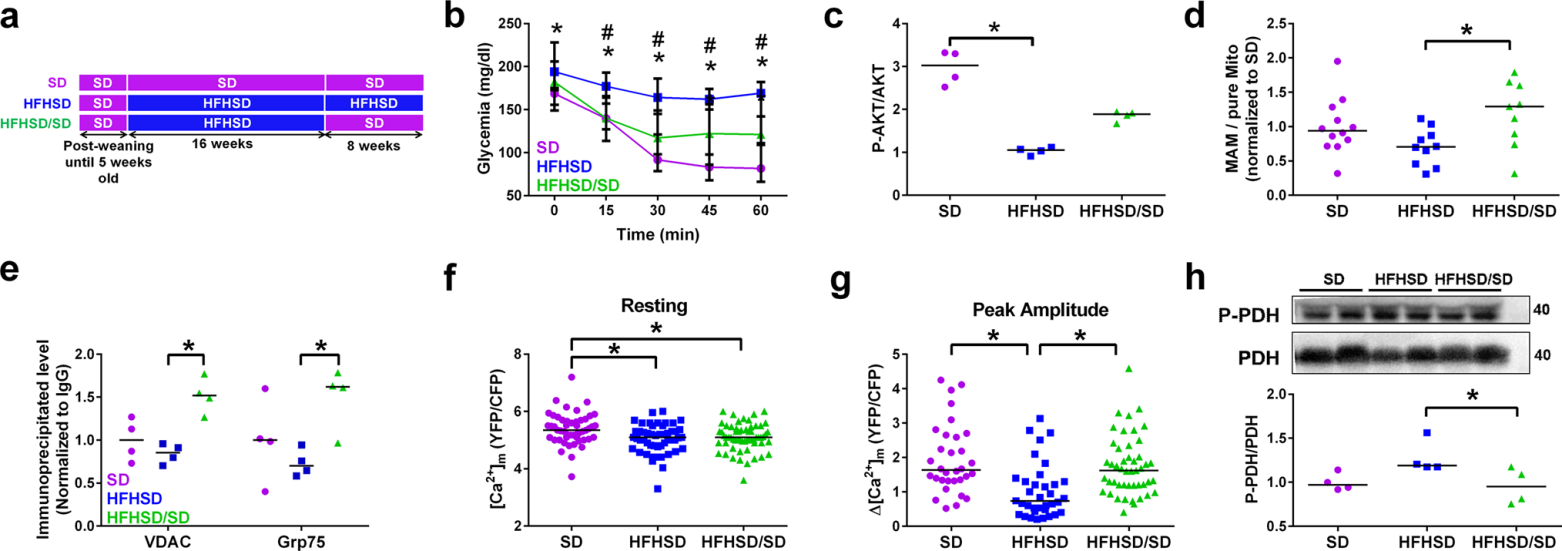
Diet reversal restores the reticulum–mitochondria functional Ca^2+^ coupling **a** Experimental design of the feeding protocols: 5-week-old mice received either 16 weeks of Standard diet (SD) or High-Fat High-Sucrose diet (HFHSD), followed by 4–8 weeks of diet reversal for a group of HFHSD mice (HFHSD/SD). **b** Insulin tolerance test following glycemia level after an intraperitoneal injection of insulin (0.75 mU/g) in mice with 4 weeks of diet reversal (*N* = 8–11 mice/group; Tukey’s Multiple comparison test). *p < 0.05 versus SD, #*p* < 0.05 versus HFHSD/SD. **c** Quantitative analysis of insulin-stimulated phosphorylation of AKT in heart of SD, HFHSD, and HFHSD/SD mice after 4 weeks of diet reversal (*N* = 4 mice/group; Kruskal–Wallis). Data are expressed as a fold of insulin stimulation over NaCl treatment. **d** Quantitative analysis of protein levels in MAM fractions following subcellular fractionation of SD, HFHSD, and HFHSD/SD hearts at 8 weeks of diet reversal. MAM amount is normalized to the pure mitochondrial protein level and expressed as a fold of SD (*n* = 9–12 mice per group). **e** Densitometric analysis of the protein amounts of VDAC and Grp75 pulled-down following IP3R immunoprecipitation in total cardiomyocyte lysates after 8 weeks of diet reversal, with normalization to IgG (*N* = 4 mice/group at 24 weeks of diet; Kruskal–Wallis). **f** Resting [Ca^2+^]_m_ values measured by the FRET-based sensor, 4mtD3cpv, in isolated cardiomyocytes following 8 weeks of diet reversal (*n* = 49–60 cells from *N* = 4 mice/group; Mann–Whitney test). **f** Summary data show the peak amplitude level of [Ca^2+^]_m_ after IP3-AM stimulation (*n* = 32–47 cells from *N* = 4 mice/group at 24 weeks of diet, Mann–Whitney test; *p* = ns when statistics made on *N* = 4 mice/group), calculated by subtracting the resting [Ca^2+^]_m_ level to the peak [Ca^2+^]_m_ level. **h** Representative immunoblotting and densitometric analysis of the phosphorylation of PDH over total PDH, relative to SD, in total cardiomyocytes lysates (*N* = 4 mice per group at 24 weeks of diet, Mann–Whitney test; *p* < 0.05 when statistics made on *N* = 4 mice/group). Results are expressed as median; * *p* < 0.05

Recent clinical studies suggest that a healthy diet might prevent worsening of T2D [30]. We questioned whether a switch to SD might reverse the T2D cardiac phenotype observed after 16 weeks of HFHSD in our mouse model. After 16 weeks of HFHSD, mice were either returned to SD (named HFHSD/SD) or kept on HFHSD (named HFHSD) for an additional 4 or 8 weeks; meanwhile, SD mice were kept on the same diet for an additional 4 or 8 weeks (Fig. 6a). As early as 4 weeks after diet reversal, HFHSD/SD mice regained near normal global insulin sensitivity as shown by the insulin tolerance test (Fig. 6b), with a partial recovery of the cardiac insulin sensitivity as shown by the enhanced insulin-stimulated Ser473-AKT phosphorylation in the heart (Fig. 6c). HFHSD/SD mice displayed a significant decrease in body weight as compared to HFHSD mice (Supplementary Fig. 5a); mean weight remained, however, significantly higher than in SD mice (Supplementary Fig. 5a).

Because of this incomplete reversal, we extended the study for 4 weeks (Supplementary Fig. 5a). Enriched MAM isolation showed a significant reversal of the protein content ratio of total MAM over pure mitochondria in the HFHSD/SD compared to HFHSD hearts (Fig. 6d). IP3R1 immunoprecipitation revealed an increased pull-down of both VDAC and Grp75 in HFHSD/SD hearts (Fig. 6e), suggesting a reconstitution of the IP3R1/Grp75/VDAC Ca^2+^ channeling complexes in these cardiomyocytes. Using our in vivo adenoviral strategy to express the mitochondrial Ca^2+^ sensor 4mtD3cpv in adult cardiomyocytes (Fig. 3d), we observed a significant twofold retrieval of the [Ca^2+^]_m_ peak amplitude in HFHSD/SD versus HFHSD cardiomyocytes and comparable to that of the SD, with no significant change at baseline (Fig. 6f-g). The rescue of mitochondrial Ca^2+^ handling was accompanied by a decreased phosphorylation of PDH in the HFHSD/SD cardiomyocytes (Fig. 6h). Following 8 weeks of SD, echocardiography showed a significant diminution of the LV mass, a reduction of the anterior and posterior wall thickness, and a recovery of the regional strain rates (Table 1). Triglycerides concentration, lipid droplets visualized by OilRedO staining (Supplementary Fig. 5b-c), and fibrosis (HFHSD: 0.43 [0.24, 0.86] vs HFHSD/SD: 0.22 [0.07, 0.33] % of tissue area, *N* = 3 mice/group, *p* < 0.05) were significantly reduced by this extended diet reversal. Altogether, these data strongly demonstrate that the T2D-induced alterations of the reticulum–mitochondrial Ca^2+^ coupling and the ensuing mitochondrial and heart dysfunctions are reversible.

## Discussion

We here report that the structural and functional alteration of the reticulum–mitochondria Ca^2+^ coupling, resulting in mitochondrial Ca^2+^ mishandling and energy disruption, is an early feature of the cardiomyopathy induced by a 16-week HFHSD in the mouse model. This HFHSD-induced cardiomyopathy is characterized by a reduced mitochondrial Ca^2+^ uptake secondary to a reduced formation of the IP3R1/Grp75/VDAC1 Ca^2+^ channeling complex in MAM but without any alteration of the mitochondrial Ca^2+^ uniporter. Moreover, the observed HFHSD-induced MAM functional Ca^2+^ uncoupling contributes to the cardiomyopathy with diastolic dysfunction and mild systolic dysfunction in the absence of any detectable change in reticular Ca^2+^ stocks, cytosolic Ca^2+^ transients, and Ca^2+^ currents at the plasma membrane. Importantly, both the abnormalities in Ca^2+^ fluxes and the structural and functional alterations of the heart are reversed following a switch back to a standard diet. Altogether, our study demonstrates that the alteration of the MAM thickness towards tighter interactions not compatible with the Ca^2+^ transport process is an early trigger of the mitochondrial dysfunctions during diabetic cardiomyopathy.

Knowing that diabetes is a metabolic syndrome that impairs many vital organs, both structurally and functionally, performing the study in vivo with a representative model of T2D, was a very crucial requisite to understand better these impairments and the mechanisms behind the altered phenotype. One major asset of this mouse model is that it recapitulates the early phase of a T2D-like cardiomyopathy, including diastolic dysfunction, LV remodeling with cardiac fibrosis, lipid accumulation, and concentric hypertrophy, together with impaired mitochondrial functions at the molecular level [39]. One must, however, acknowledge that our HFHSD mice did not reach the heart failure stage with marked lipotoxicity and oxidative stress, but rather correspond to the early stages of development of the diabetic cardiomyopathy, with subtle systolic dysfunction. In front of the lack of cultured cardiac cell models which fulfill all the characteristics of DCM [18], we have taken advantage of our in vivo intramyocardial delivery of adenoviruses encoding different Ca^2+^ sensors to evaluate the early mechanisms of DCM in adult cardiomyocytes freshly isolated from HFHSD mice.

Previous works have demonstrated a reduced mitochondrial Ca^2+^ uptake with a decreased mitochondrial ATP production in the diabetic cardiomyocyte [16, 20, 34], but the trigger of these alterations had not yet been elucidated. We speculated that a modification either at the level of the mitochondrial Ca^2+^ entry via the mitochondrial Ca^2+^ uniporter or even upstream at the MAM functional Ca^2+^ coupling would lead to this mitochondrial Ca^2+^ mishandling. We demonstrated here, by combining structural and functional analysis of the reticulum–mitochondria Ca^2+^ coupling, a reduced formation of the IP3R1/Grp75/VDAC1 Ca^2+^ channeling complex in the MAM interface of HFHSD cardiomyocytes due to tighter reticulum–mitochondria interactions and MAM protein composition rearrangement, leading to subsequent reduced Ca^2+^ transfer from the reticulum to mitochondria. While no change in the reticular Ca^2+^ stock was measured, a small increase in the resting cytosolic Ca^2+^ content was observed, suggesting a redirection of the reticular Ca^2+^ fluxes toward the cytosol instead of mitochondria. On a long term, the reticular Ca^2+^ efflux may become excessively leaky contributing, therefore, to cytosolic and mitochondrial Ca^2+^ overload and heart failure progression, as previously demonstrated [51, 54]. Mitochondrial Ca^2+^ signaling plays a preponderant role in providing the energy required for the cardiomyocyte function, through the activation of the Ca^2+^-sensitive matrix dehydrogenases and the ensuing mitochondrial oxidative phosphorylation-driven ATP production [38, 40]. Recently, Seidlmayer et al. proposed that mitochondrial Ca^2+^-driven ATP production is mediated by agonist-induced IP3R-mediated Ca^2+^ release to mitochondria to match the energetic needs of the cardiomyocyte [57]. In our HFHSD mouse model, MAM functional Ca^2+^ uncoupling leads to an insufficient mitochondrial bioenergetics to match the energy demand required for normal heart contraction. Using a MAM linker expression strategy in HFHSD mice that reversed the Ca^2+^ and contractile dysfunction, we established the causative upstream role of chronic reticulum–mitochondria Ca^2+^ miscoupling on mitochondrial Ca^2+^ mishandling and the ensuing cardiomyocyte contractile dysfunction. Therefore, our data point out to the reduced mitochondrial bioenergetics and energy depletion, due to a decreased Ca^2+^ transfer from reticulum to mitochondria, as a leading cause behind the altered cardiac function. Our OCR data in intact versus permeabilized cardiomyocytes, a relevant protocol to assess mitochondrial bioernergetics [13, 47], support that the decreased mitochondrial bioenergetics in the HFHSD cardiomyocytes do not rely on alteration of the electron transport chain but rather on the deficient reticulum-mitochondria Ca^2+^ transfer. Indeed, a similar mitochondrial membrane potential was measured in both SD and HFHSD isolated cardiac mitochondria, confirming the good functional state of the isolated HFHSD mitochondria. Several studies have reported a decreased ATP content (up to 34%) associated with a subtle, but not severe, systolic dysfunction [29, 34, 70]. Interestingly, the reduced maximal ATP production and total ATP content that we measured is in keeping with the subtle systolic dysfunction measured by echocardiography. We should also consider that compensatory increase in heart rate and the observed myocardial hypertrophy might have contributed to attenuate the apparent contractile dysfunction.

**Fig. 7:**
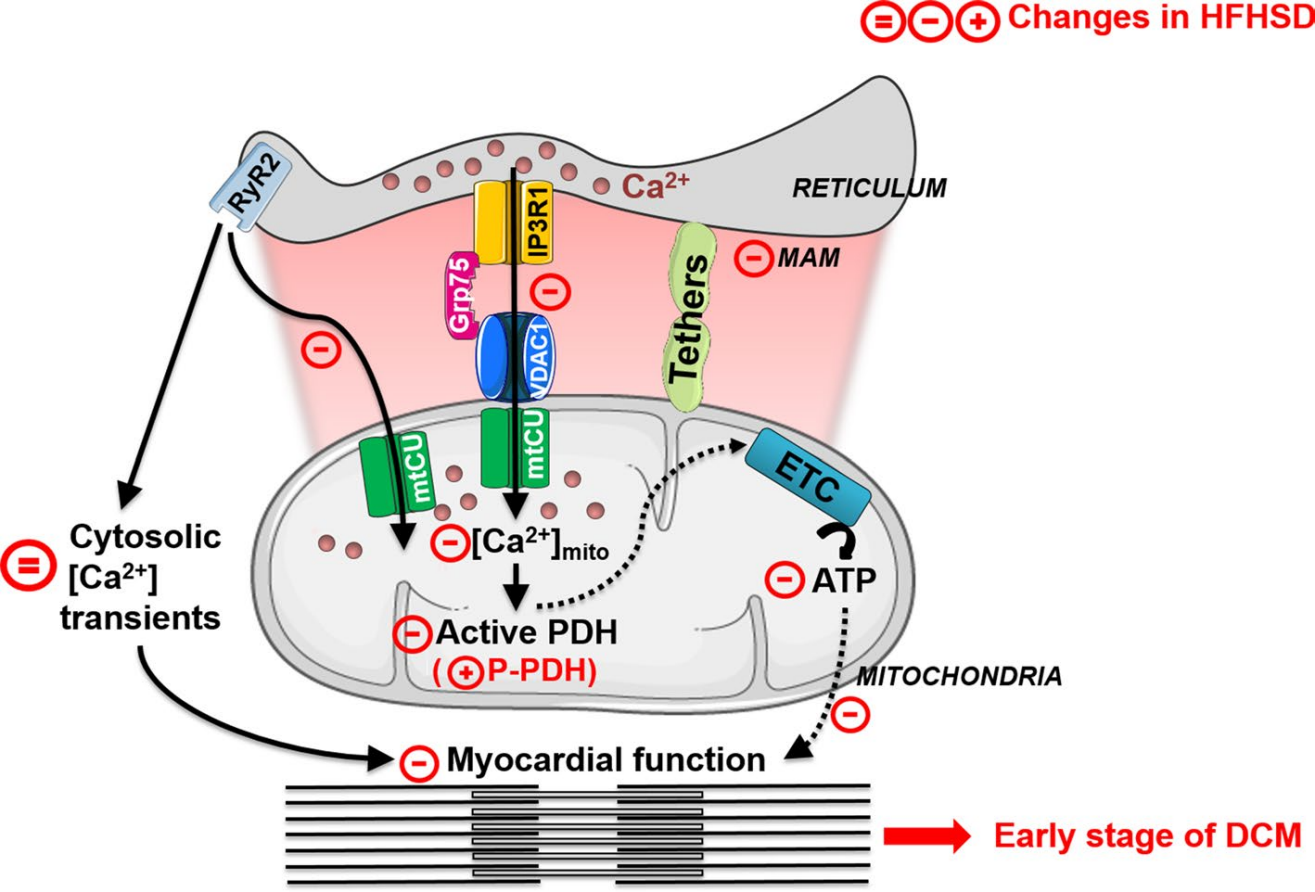
Contribution of the reticulum–mitochondria functional Ca^2+^ disruption and the ensuing mitochondrial dysfunctions to the pathophysiological mechanisms of type 2 diabetes-induced contractile dysfunction. During physiological excitation–contraction coupling, reticular Ca^2+^ release through the Ryanodine receptor (RyR) leads to cytosolic Ca^2+^ transients. In parallel, Ca^2+^ transfer from reticulum to mitochondria through both RyR and IP3R at the MAM interface increases the mitochondrial [Ca^2+^], therefore activating the PDH and the ATP production through the electron transport chain (ETC). Both cytosolic Ca^2+^ elevations and ATP activate concomitantly the contraction. In conditions of HFHSD-induced type 2 diabetes, the cardiac reticulum–mitochondria functional Ca^2+^ coupling is disrupted with a decreased formation of the IP3R/Grp75/VDAC Ca^2+^ channeling complex, thus leading to a reduced transfer of Ca^2+^ from the reticulum to mitochondria. In parallel, a decreased mitochondrial Ca^2+^ uptake under field stimulation is seen in the HFHSD model, with a preservation of cytosolic Ca^2+^ transients. This reduction in mitochondrial Ca^2+^ level contributes to the occurrence of mitochondrial dysfunctions, notably a decreased PDH activity followed by a decreased mitochondrial bioenergetics. Consequently, the insufficient ATP content fails to match the energy demand for the cardiomyocyte contraction. Therapeutic diet reversal counteracts the disruption of the reticulum–mitochondria functional coupling, therefore, reestablishing the proper mitochondrial Ca^2+^ handling and metabolic function, required for the normal functioning of the heart.

Based on these previous studies and the present report, we propose the following contribution of the reticulum–mitochondria functional Ca^2+^ miscoupling and the ensuing mitochondrial dysfunctions to the pathophysiological mechanisms of type 2 diabetes-induced contractile dysfunction (Fig. 7). The initial disruption of the reticulum–mitochondria functional Ca^2+^ coupling by loss of Ca^2+^ transport compatible-tethering partners (like MFN2) towards tighter junctions contributes to the occurrence of mitochondrial dysfunctions, notably a decreased PDH activity followed by a reduction of mitochondrial respiration through the electron transport chain. This insufficient ATP content fails to match the energy demand required for the cardiomyocyte contraction.

Several studies have shown a reduction of MAMs Ca^2+^ coupling in most of T2D organs, including liver [50, 66], kidney [62], pancreatic beta cells [62], skeletal muscle [65], and brain [35]; while increased MAMs Ca^2+^ coupling was reported in diabetic liver and skeletal muscle by two other groups [1, 63]. The width of the reticulum–mitochondria junction is now well recognized as a structural parameter under tight regulation to control and define its function [2, 17]. Owing to the complex and rather rigid ultrastructure of the reticular and mitochondrial networks in the adult cardiomyocytes, one may expect that the MAM modulation rather relies on changes in thickness than in length. Indeed, our ultrastructural analysis of the cardiac reticulum–mitochondria interactions suggests that the MAM interface is rather stable in the heart, since we did not obtain any difference in the interface length. However, we detected significant alterations of the MAM thickness, which is critical for each MAM function. Interestingly, our ultrastructural and proteomic analysis of cardiac MAM revealed in the HFHSD MAM: (1) tighter interactions in the 0–10 nm range together with increased expression of proteins involved in lipid metabolism process, and (2) decreased interactions at 20–30 nm with decreased protein expression in the ion transport and ATP metabolic processes. These data further support our functional analysis showing a decrease in the reticulum–mitochondria Ca^2+^ transfer and the mitochondrial bioenergetics, and suggest a redirection of MAM function toward a lipotoxic effect. Therefore, our study strongly supports the importance of analyzing not only the length but rather the thickness of the MAM interface, which could be one explanation for the observed discrepancies cited above. Recent reports in cardiomyocytes also indicated an altered mitochondrial Ca^2+^ uniporter in the db/db mice [23] and increased MAMs in a type 1 diabetic animal model [69], both models of late diabetes showing an increased mitochondrial Ca^2+^ uptake with strong systolic dysfunction. Obviously, obesity, type 1, and type 2 diabetes are distinct metabolic diseases with different molecular mechanisms and cardiac phenotypes [15, 39]. Moreover, these discrepancies between groups and organs could also be related to the timing of the diabetic alterations. Indeed, our type 2 diabetic mouse model is a nutritional model which phenocopies well the “common” obesity and is, therefore, closer to the physiopathology of the human diabetes with early phenotype of cardiomyopathy, i.e., mitochondrial alterations with mild oxidative stress, and diastolic dysfunction without reaching heart failure. On the other hand, the genetic ob/ob and db/db mouse models correspond to “monogenic” obesity, which only refers to a small percentage of the human diabetic population. Of note, while db/db mice are a model of advanced DCM, the ob/ob model is rather an early one as our HFHSD model [7]. Interestingly, we, using the HFHSD model, and Fauconnier et al., with the ob/ob mice [16], both reported a reduced IP3-driven mitochondrial Ca^2+^ transfer, suggesting that the timing of the diabetes may be more important than the diabetic origin, to take into account for the study of the underlying mechanisms. Since MAMs are dynamic entities, one could wonder whether the progression of diabetes is accompanied by a maladaptive compensation, such as increased MAMs’ coupling to improve the Ca^2+^ transfer towards the risk of increased cell death. Additionally, we did not find any alteration in mitochondrial uniporter function in our model at that stage, suggesting that changes in uniporter function and composition may occur later, notably for the compensation of the reduced [Ca^2+^]_m_. Future studies will be required to decipher between these differences. Nonetheless, our results of disrupted MAMs Ca^2+^ coupling in the heart are in line with these studies and suggest a systemic effect of T2D. Additional work is needed to clarify the impact of cardiac insulin resistance on the reticulum–mitochondria interface structure and function. Our proteomic analysis of the cardiac MAM proteome clearly highlights a change in the protein composition by the HFHSD. The protein recruitment at the MAM might involve post-translational modifications, such as glucotoxicity-associated O-*N*-acetylglucosamine acylation as previously suggested [22, 36] or changes in the lipid composition of the MAM, since previous studies in diabetic mice demonstrated that HFHSD impacted mitochondrial liver phospholipid composition [67] and the cardiolipin remodeling in the diabetic myocardium [21]. Future lipidomics analysis of the diabetic cardiac MAM will help to unravel the initial trigger of the protein composition rearrangement of the cardiac MAM under T2D.

While our findings support that the decreased reticulum-driven mitochondrial Ca^2+^ uptake, consequently leading to reduced mitochondrial Ca^2+^ content, is an early trigger of mitochondrial dysfunctions in diabetic cardiomyopathy, an intriguing point is raised by the absence of a significant role for MCU in baseline cardiac function using an acute MCU-KO mouse [33]. Indeed, Luongo et al. reported no change in baseline cardiac structure and function, as well as mitochondrial bioenergetics by acute MCU deletion; however, they showed that MCU is required for matching the mitochondrial energetic production to the contractile demand during the fight-or-flight response [33]. Importantly, the MCU-KO cardiomyocytes did not present any change in mitochondrial Ca^2+^ content, contrary to our HFHSD model, which could be due to an MCU-independent and compensatory mechanism of mitochondrial Ca^2+^ uptake, not yet identified. Therefore, contrary to the acute MCU-KO mouse, our HFHSD model consists in a chronic downregulation of mitochondrial [Ca^2+^], pointing out the mitochondrial Ca^2+^ dysregulation following the reticulum–mitochondria miscoupling as a main contributor to the development of DCM. Additionally, recent studies also demonstrated that mitochondrial bioenergetics is not only regulated by mitochondrial Ca^2+^ but also by cytosolic Ca^2+^ via the malate-aspartate shuttle [27, 59], and that there is a feedback between metabolite flux and mitochondrial Ca^2+^ uptake [41, 64], highlighting the complex relationship between mitochondrial metabolism and Ca^2+^. We cannot exclude that this mechanism participates in the reduced mitochondrial Ca^2+^ content of HFHSD mice.

A major observation of our study is that an 8-week switch back to a standard diet reestablishes the functional Ca^2+^ coupling of the reticulum–mitochondria interface and a normal Ca^2+^ transfer in the HFHSD heart; consequently, baseline cardiac function of the heart is recovered. This first indicates that Ca^2+^ transfer from reticulum to mitochondria through MAM to maintain a physiological resting mitochondrial [Ca^2+^] is a central mechanism of the T2D cardiomyopathy and that these initial modifications of the heart are reversible. Furthermore, this strongly supports the idea that optimization of the diet might be a very important component of the therapy aimed to attenuate the early progression of cardiomyopathy in T2D patients.

In summary, we propose that the transfer of Ca^2+^ through MAMs is an early and crucial player of the mitochondrial Ca^2+^ mishandling and dysfunction in T2D hearts. Our work further suggests that adaptation of patients’ nutrition might prevent these early modifications and subsequently counteract the progression or even reverse the associated cardiomyopathy.

## Limitations

While being an invaluable asset for the study of in vivo T2D alterations, analysis in freshly isolated adult mouse cardiomyocytes knows some limitations. Intramyocardial adenoviral infection is leading to a maximum of 30% of infected cardiomyocytes. Therefore, this point precluded any further in vivo analysis of cardiac function and non-single cells targeted measurements, such as electron microscopy, OCR, and immunoblotting, since we could not select the infected cardiomyocytes. Moreover, our diet-induced T2D mouse model also triggers obesity, which prevents us from deciphering between diabetes and obesity-related effects. Finally, the 4mtD3cpv FRET-based sensor of mitochondrial Ca^2+^, due to its Kd of 0.6 µM, is not the most adequate to assess both changes in the resting mitochondrial Ca^2+^ and in the IP3-induced peak amplitude. Higher magnitude of changes are assessed in the peak level; nevertheless, small magnitude changes in resting levels are still of biological meaningful difference.

## Conclusion

Diabetic cardiomyopathy is a very common complication in type 2 diabetes, notably as a result of mitochondrial dysfunction ending by a compromised cardiac function. Our study shows that alteration of the reticulum–mitochondria Ca^2+^ coupling impairs mitochondrial Ca^2+^ stocks and bioenergetics in the diabetic heart, further altering the myocardial function, which could be reversed by diet reversal. Interestingly, the control of nutritional intake showed its direct contribution in the improvement of cardiac function in the early stage of diabetic cardiomyopathy, specifying the underlying mechanisms behind the beneficial effect of diet reversal.

## Electronic supplementary material

Below is the link to the electronic supplementary material.

Supplementary file1 (PDF 532 kb)

Supplementary file2 (PDF 1890 kb)

## Data Availability

The datasets used and/or analyzed during the current study are available from the corresponding author on reasonable request. Proteomic data are available via ProteomeXchange with identifier PXD015280.
