# Supplementary material for: Reduced reticulum–mitochondria Ca2+ transfer is an early and reversible trigger of mitochondrial dysfunctions in diabetic cardiomyopathy
Source: Basic Res Cardiol. 2020 Nov 30;115(6):74. doi: 10.1007/s00395-020-00835-7 (PMC7704523; doi:10.1007/s00395-020-00835-7)
Supplement: Supplementary file 1 — Supplementary file1 (PDF 532 kb) [file 395_2020_835_MOESM1_ESM.pdf]

SUPPLEMENTARY MATERIALS

Reduced reticulum-mitochondria Ca^{2+} transfer is an early and reversible trigger of mitochondrial dysfunctions in diabetic cardiomyopathy

Maya Dia[1, 2], Ludovic Gomez[1], Helene Thibault[1, 5], Nolwenn Tessier[1], Christelle Leon[1], Christophe Chouabe[1], Sylvie Ducreux[1], Noelle Gallo-Bona[1], Emily Tubbs[3], Nadia Bendridi[3], Stephanie Chanon[3], Aymeric Leray[4], Lucid Belmudes[5], Yohann Couté[5], Mazen Kurdi[2], Michel Ovize[1, 6]*, Jennifer Rieusset[3,]*, Melanie Paillard[1]

* Contributed equally to the work

Correspondence:

Dr. Melanie Paillard

Laboratoire CarMeN-équipe 5 Cardioprotection, INSERM, INRA, Université Claude Bernard Lyon-1, INSA-Lyon, Univ-Lyon

Groupement Hospitalier Est, Bâtiment B13

59 boulevard Pinel

69500 BRON, FRANCE

Tel: +33 (0)4.78.78.56.10

E-mail : melanie.paillard@univ-lyon1.fr

SUPPLEMENTARY METHODS

Heart Mitochondria Isolation

After euthanasia of the animal by cervical dislocation, the heart was removed and placed in a beaker with isolation buffer (225 mM Mannitol, 75 mM Sucrose, 20 mM HEPES, 0.1 mM EGTA) on ice. Then it was washed, dried and weighed. The heart was minced to very small pieces then poured into a glass tube. Isolation Buffer with BSA (4.5 ml) was then added, followed later by the addition of proteases (3U/g tissue) inside the potter directly for 1 min incubation.

Homogenization was performed (10 strokes/300rpm with the Teflon pestle), then the homogenate was transferred into a 15ml Falcon tube and centrifuged at 500 x g for 5 min at 4°C to pellet nucleus, debris and plasma membranes. The supernatants were transferred into 3 eppendorf tubes and centrifuged at 9000 x g for 10 min at 4°C.

The mitochondria pellet was finally resuspended with 100 µl resuspension buffer (225 mM mannitol, 75 mM sucrose, 10 mM HEPES) and protein quantified by the Lowry method.

Fluorometric Measurements of Mitochondrial Ca^{2+} Uptake and Membrane Potential

As previously described [6], 500 µg mouse heart mitochondria were resuspended at 35°C in 1.5 ml of an intracellular medium, ICM (120 mM KCl, 10 mM NaCl, 1 mM KH_2PO_4, 20mM HEPES/Tris, pH 7.2) supplemented with 2 mM Mg-ATP, 2 µM Thapsigargin, protease inhibitor cocktail (Sigma, P8340) and 20 µM CGP-37157. The ratiometric Ca^{2+} probe Fura 2-FA (1.5 µM) and Fura-FF (1 µM, INTERCHIM, FP-AM627B) were used to assess respectively the extramitochondrial low (3 and 7 µM) and high (10 and 50 µM) Ca^{2+} concentrations $[Ca^{2+}]_c$, while mitochondrial membrane potential was recorded with 1 µM of TMRM (Thermo Fischer, T668) in a Hitachi F2500 spectrofluorometer. Fluorescent signals of Fura and TMRM were recorded using a 340-380 excitation/500 emission, and 545 ex/580 em respectively. The protonophore FCCP (2 µm) was then used to elicit a maximal

depolarization. The Fura signal was calibrated 1 mM $CaCl_2$ followed by 10 mM EGTA/Tris (pH 8.5).

Mouse adult cardiomyocytes isolation

After mouse euthanasia by cervical dislocation, cardiomyocyte isolation was performed by collagenase digestion following O'Connell's protocol [4]. Cardiomyocytes were then plated on laminin-coated glass coverslips for live imaging or 8-wells chamber slides for PLA.

Proximity Ligation Assay (PLA)

Cardiomyocytes were fixed with 4% paraformaldehyde (10 minutes at room temperature) and permeabilized with 0.1% Triton X-100 (15 minutes at room temperature). PLA protocol was performed according to recommendations from manufacturers (Sigma) and as previously described [7]. Primary antibodies used were: IP3R1 (1/200, sc28614), VDAC (1/200, ab14734) and MCU (1/200, HPA016480). Image acquisition was done using laser scanning confocal microscope (Nikon A1R, 60x objective) with $\lambda_{ex/em}$=401.8/450 nm for DAPI and $\lambda_{ex/em}$=560.8/595 nm for red fluorescent dots. Dots quantification was performed by the NIS elements software with one identical threshold set for all images.

Cardiomyocyte area quantification

Cardiomyocyte area was quantified using ImageJ software on the Ca^{2+} imaging pictures.

Mitochondrial respiration in permeabilized cardiomyocytes

300µg of cardiomyocytes were suspended in mitochondrial respiration medium containing 50 mM Tris-HCl, 100 mM KCl, 5 mM KH2PO4, 1 mM EGTA (pH 7.4), and 0.1% BSA. To determine the oxygen consumption rates of the different ETC complexes, a successive substrate-uncoupler inhibitor titration protocol was performed on a high-resolution oxygraph (Oxygraph-2k; Oroboros, Innsbruck, Austria) at 25°C. After 2 minutes of permeabilization with digitonin (40µM) and in presence of glutamate/pyruvate/malate (5mM each), state 2 was

measured. Addition of 2 mM ADP led to the measurement of OCR through complex I. Successive additions of rotenone (1.25 µM), +succinate (complex II substrate; 5 mM), followed by antimycin A (12.5 µM) and TMPD/ascorbate (complex IV substrates; 0.125 and 1.25 mM respectively) allowed determination of sensitive rates of oxidative phosphorylation using complexes II and IV substrates, respectively. Data were analyzed with the Oroboros DatLab4 software and expressed as nanomoles of oxygen per minute per milligram of proteins.

Electron microscopy

For ultrastructural study, isolated cardiomyocytes in-suspension were fixed with 2% glutaraldehyde (EMS) in 0.1 M sodium cacodylate (pH 7.4) buffer. After washing three times in 0.2 M sodium cacodylate buffer, cell cultures were post-fixed with 1% aqueous osmium tetroxide (EMS) for 1 hour and deshydrated in a graded series of ethanol at room temperature and embedded in Epon. After polymerization, ultrathin sections (100 nm) were cut on a UC7 (Leica) ultramicrotome and collected on 200 mesh grids. Sections were stained with uranyl acetate and lead citrate (EMS) before observations on a Jeol 1400JEM (Tokyo,Japan) transmission electron microscope equipped with an Orius 600 camera and Digital Micrograph. This microscope is located at the CIQLE platform (Centre d'Imagerie Quantitative Lyon Est, France). Interfaces between reticulum and mitochondria were blindly analyzed using a custom Image J plugin/macro, as previously published [1].

Blood pressure

Blood pressure was assessed in anesthetized mice (2.5-3.5% Sevoflurane) using a non-invasive tail-cuff system (Coda, Kent Scientific).

Histology

Hearts were fixed with 4% paraformaldehyde and then paraffin-embedded at the CIQLE imaging Platform (Lyon-France) to perform a Masson's Trichrome staining. Sections were

observed using the Zeiss AxioScan Z1 slide scanner. Images were blindly analyzed using the ZEN software and ImageJ with a macro from Denis Resnikoff.

For oil red O staining (kit ORO-k-250, Biognost), hearts were embedded in Tissue-Tek-OCT and 10µm-cryosections were performed. Analysis was performed using a custom-written Fiji macro.

Triglycerides

Total triglycerides content was evaluated from mouse heart lysate using an enzymatic kit (Biolabo, Maizy, France).

Total ATP levels

Total ATP content was detected using an ATP Bioluminescence Assay Kit (Roche 11699709001) on freshly isolated adult cardiomyocytes at 16 weeks of diet.

ROS assessment

Cardiomyocytes were incubated with 2µM MitoSOX red (M36008) for 10 min/37°C in suspension. Mitochondrial superoxide ion levels were assessed by flow cytometry using a BD Fortessa-X20 flow cytometer, as previously described [5]: results were expressed as a percentage of positively-stained population.

Protein carbonylation (OxyBlot)

Oxyblot^{TM} Protein Oxidation Detection Kit (Millipore S7150) was used to derivatize the proteins (1X DNPH for 15 min) from cardiomyocyte lysates prepared in complete RIPA lysis buffer. 20 µg of total proteins were separated on a 10% SDS-PAGE gel and subjected to western blotting with anti-DNP antibody. Blots were scanned and protein carbonylation level normalized to the Coomassie blue staining gel.

Patch Clamp Electrophysiology

Current recordings were made at room temperature under voltage clamp using the whole-cell configuration of the patch-clamp technique. Command voltage and data acquisition were performed with pClamp software (Axon Instruments, Foster city, CA, USA). The L-type Ca^{2+} current ($I_{Ca,L}$) was evoked every 10 s by 250-ms voltage steps spaced 10 mV apart and varying between -90 to 60 mV and was measured as the difference between the peak inward current and the current at the end of the pulse. The measurement of the Na^{+}-Ca^{2+} exchange current (I_{NCX}) was made as described by Espinosa et al. [3]. The holding potential was kept at -80 mV. Membrane capacitance was systematically measured and was calculated by analyzing the capacitive surge produced by a small voltage step as previously described [2]. Current traces were uncorrected from the leak and normalized to the capacitance. For $I_{Ca,L}$ recording, the external solution contained (in mM): 136 TEACl, 2 $MgCl_2$, 1.8 $CaCl_2$, 5 4-aminopyridine, 10 glucose, 10 Hepes, adjusted to pH 7.4 with TEAOH and the internal solution contained (in mM): 140 CsCl, 1 $MgCl_2$, 3 MgATP, 10 EGTA, 10 Hepes, adjusted to pH 7.2 with CsOH. For I_{NCX} recording, the external solution contained (in mM): 136 NaCl (or LiCl), 5 CsCl, 2 $MgCl_2$, 1.8 $CaCl_2$, 10 glucose, 5 Hepes, adjusted to pH 7.4 with NaOH (or LiOH) and the internal solution contained (in mM): 7 NaCl, 20 CsCl, 110 Cs-aspartate, 1.1 $MgCl_2$, 0.2 EGTA, 5 Hepes, adjusted to pH 7.2 with CsOH.

SUPPLEMENTARY FIGURES AND TABLE

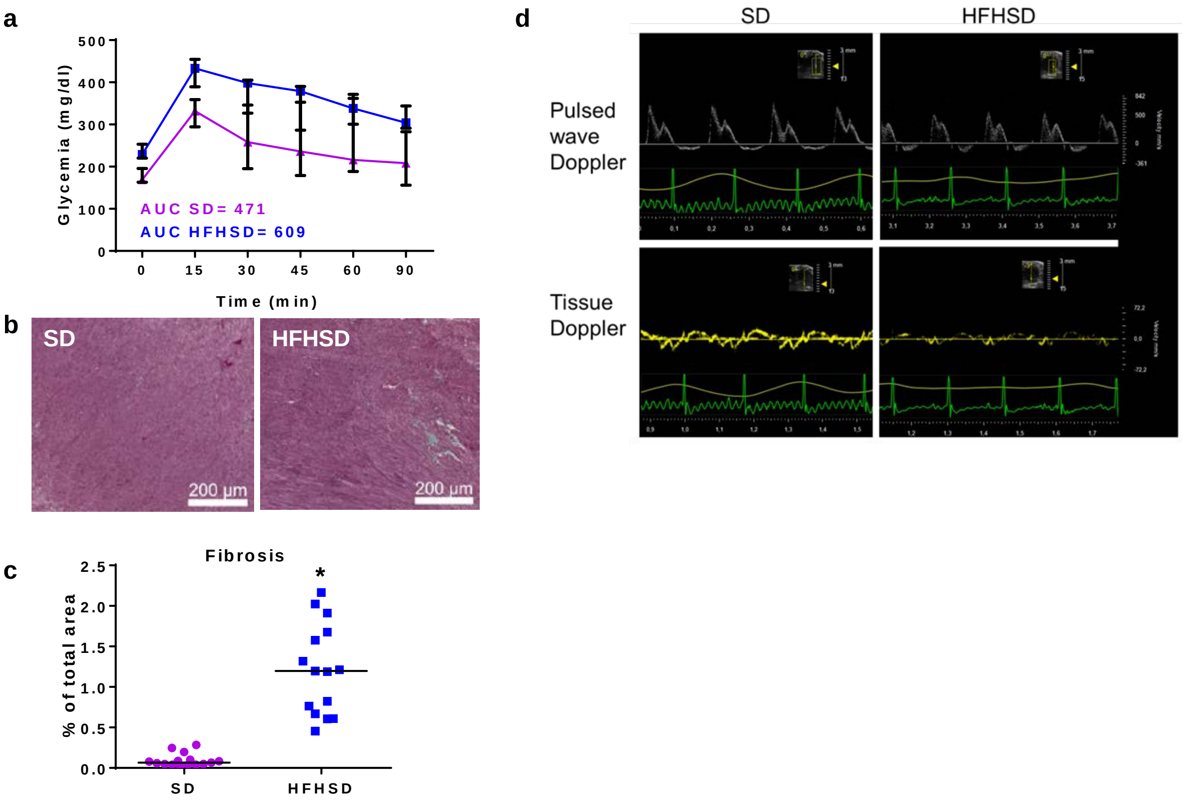

Supplementary Fig. 1, related to Fig. 1 Phenotypic characterization of the HFHSD mice. **a** Glucose tolerance assessment by measuring glycemia levels after an intraperitoneal injection of glucose (2mg/g) (N=5 mice per group). AUC: area under curve. **b** Representative images of Masson's trichrome staining of SD and HFHSD left ventricular tissue to evaluate collagen deposition (in blue). **c** Quantification of fibrotic area as a percentage of left ventricle using Zen Software (n=15 random regions being analyzed from N= 3 mice/group; Mann-Whitney test). Scale bar: 200µm. **d** Representative echocardiography images from Vevo 3100 imaging system in SD and HFHSD mice at 16 weeks. Pulsed wave Doppler was used to assess flow through the mitral valve for measurements of E and A peak velocities, and IVRT. Tissue Doppler allows E' peak velocity measurement by assessing tissue motion at the mitral valve annulus.

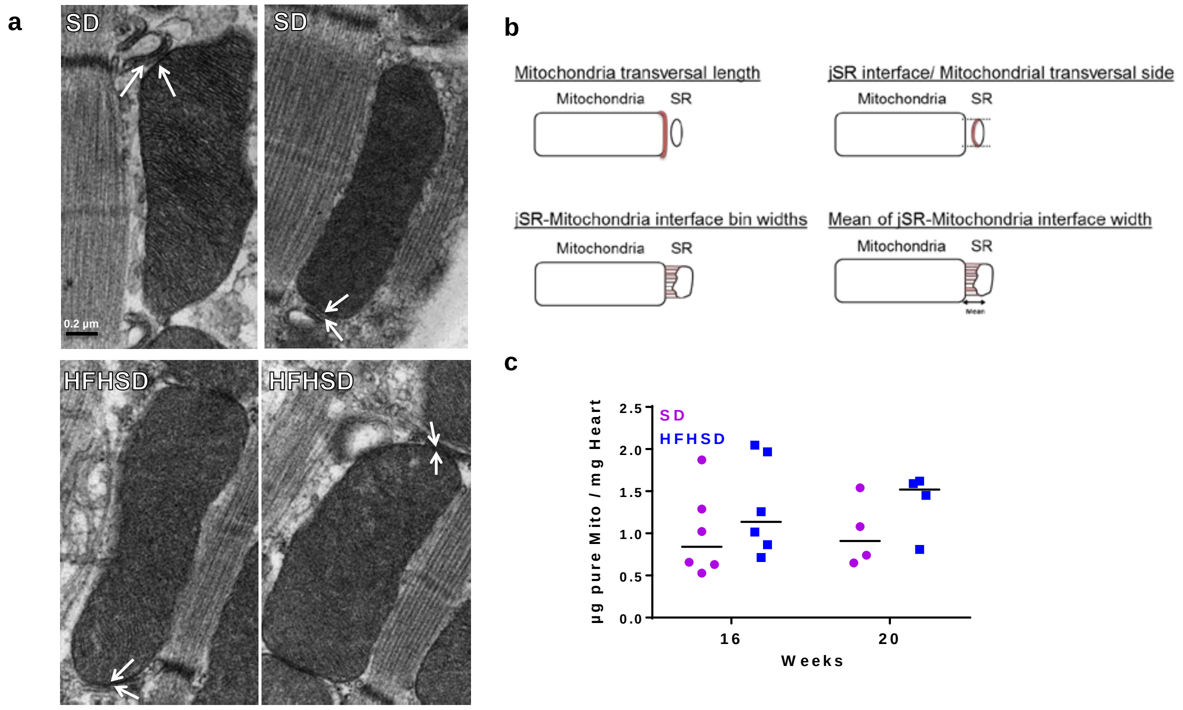

Supplementary Fig. 2, related to Fig. 2 Ultrastructural analysis of the cardiac MAM.

a Representative images of transmission electron microscopy performed on isolated cardiomyocytes from SD and HFHSD mice at 16 weeks of diet. Arrows indicate reticulum-mitochondria interface. **b** Schematics of the different parameters measured on EM images and presented in Fig.2. **c** Quantification of the pure mitochondrial protein levels normalized to heart weight, relative to SD, at 16 and 20 weeks of diet (N=4-6 mice per group; Mann-Whitney test). Results are shown as median.

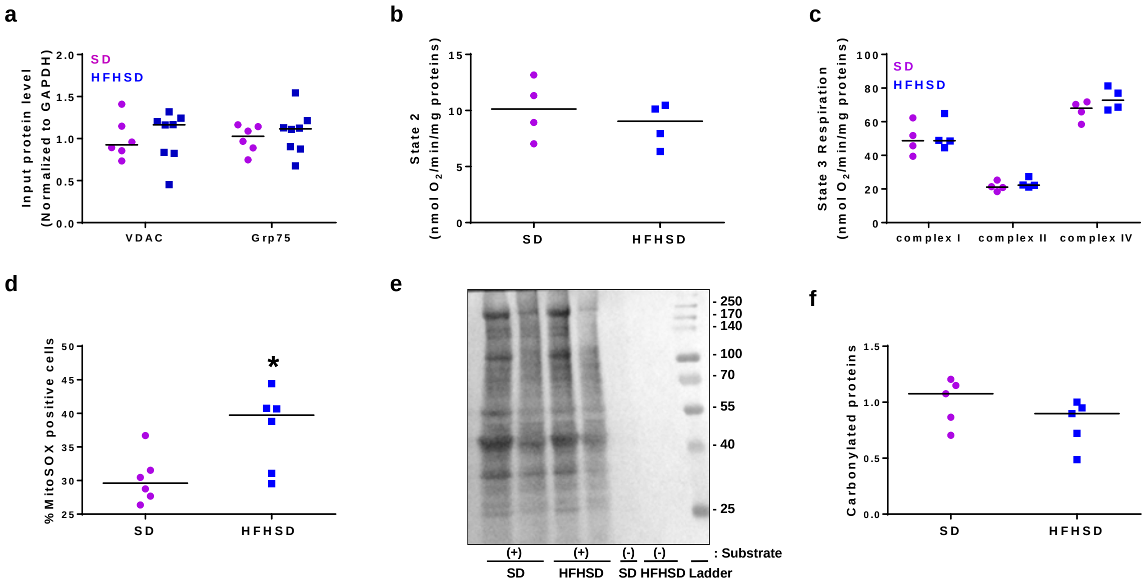

Supplementary Fig. 3, related to Fig. 3 16 weeks of HFHSD increased mitochondrial ROS production, without whole-cell oxidative stress**. a** Quantification of the VDAC and Grp75 protein levels in the total cardiomyocyte lysate (Input before IP3R IP). (N=5-8 mice/group at 16 weeks of diet; Mann-Whitney test). **b-c** Oxygen consumption rates in digitonin-permeabilized cardiomyocytes: state 2 (**b**) and state 3 (**c**) rates under complex I (glutamate/pyruvate/malate), complex II (succinate+ rotenone) and complex IV substrates (TMPD-ascorbate+antimycin A). (N=4 mice/group at 16 weeks of diet; Mann-Whitney test). **d** Percentage of MitoSOX-positive cells reflecting O_2^- levels in isolated cardiomyocytes (N=6 mice per group; Mann-Whitney test). **e** Representative immunoblotting of carbonylated proteins from SD and HFHSD isolated cardiomyocytes. **f** Densitometric analysis of the carbonylated proteins normalized to the Coomassie blue staining gel, relative to SD (N=5 mice per group; Mann-Whitney test).

Data are displayed as median.

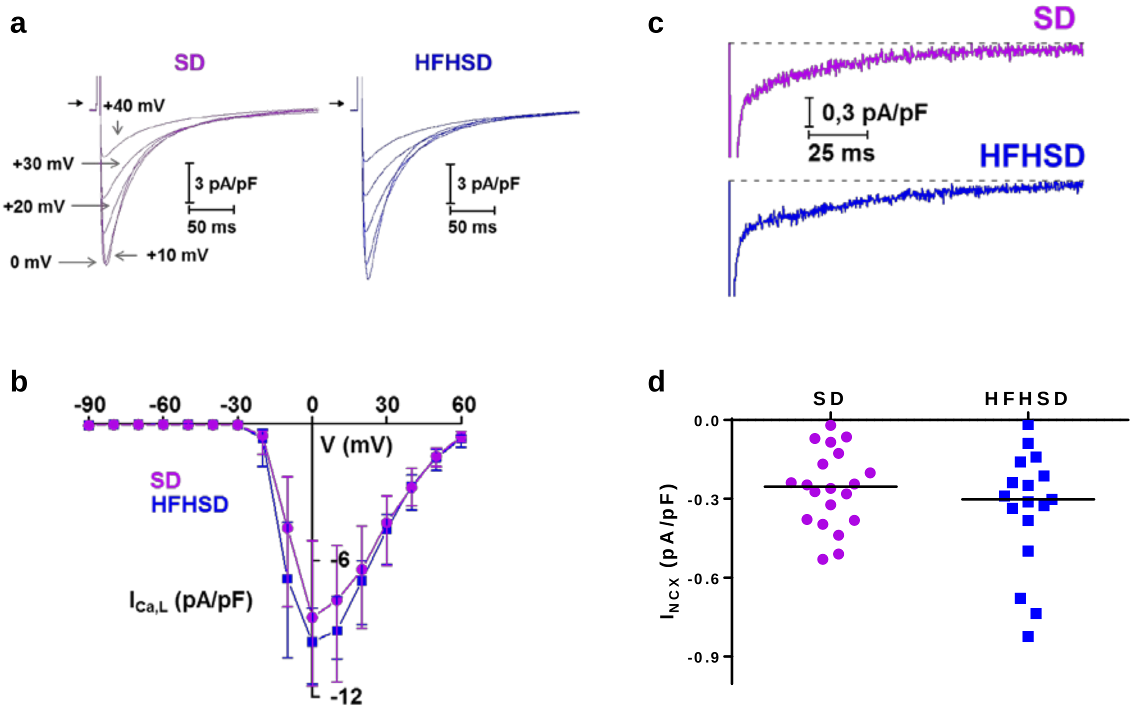

Supplementary Fig. 4, related to Fig. 4 Electrophysiological measurements of L-type Ca^{2+} and Na^{+}-Ca^{2+} exchange currents in isolated cardiomyocytes. **a** Representative traces of inward L-type Ca^{2+} current in SD and HFHSD cardiomyocytes during depolarizing steps spaced 10 mV apart and varying between 0 and +40 mV from a holding potential of -80 mV. **b** Current-voltage relationships of normalized $I_{Ca,L}$ peak to membrane capacitance from SD and HFHSD cells. Data are expressed as medians with interquartile ranges (n=20 cardiomyocytes from N=4 mice/group; Mann-Whitney test). **c** Representative traces of Na^{+}-Ca^{2+} exchange current (I_{NCX}) in SD and HFHSD cardiomyocytes. I_{NCX} was measured as the lithium-sensitive slow tail current 20 ms after the onset of the repolarization to -80 mV. **d**, Dot plot shows density of I_{NCX} and bar values are medians (n=17-20 cells from N=4 mice/group with; Mann-Whitney test).

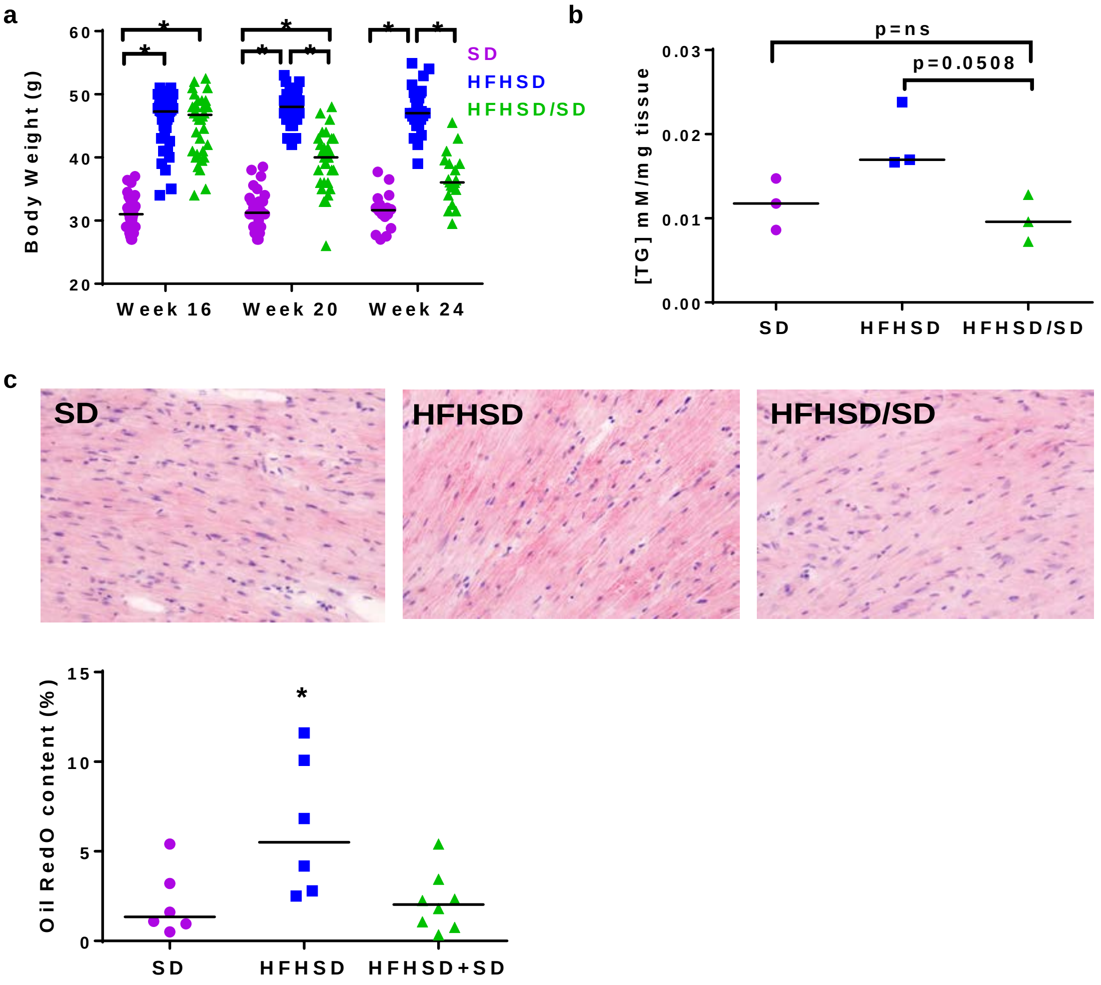

Supplementary Fig. 5, related to Fig. 6 Diet reversal effects on mouse body and heart weights, and lipid deposition. **a** Body weight measurement at the day of diet reversal launching (16 weeks of diet), and at 20 and 24 weeks, i.e. at 4 and 8 weeks of diet reversal (N=13-45 mice per group; Tukey's Multiple comparison test). **b** Quantification of triglycerides concentration in cardiac tissues (N=3 mice per group; Kruskal-Wallis). **c** Representative images and quantification of lipid deposit by OilRedO staining of cardiac tissues: lipid droplets are seen as red dots (N=6-8 mice per group). Scale bar= 50 µm. Results are displayed as median,* p<0.05.

Gene Name	Protein Name	# Peptides	Enrichment (log_2 Fold change HFHSD vs SD)	p Value
Dsp	Desmoplakin	12	1.8	6.59E-03
Ndrg2	Protein NDRG2	4	1.8	8.26E-03
Tpm1	Tropomyosin alpha-1 chain	3	1.5	1.37E-02
Jup	Junction plakoglobin	10	1.5	1.71E-02
Fabp4	Fatty acid-binding protein, adipocyte	3	1.4	6.31E-03
Dsg1a	Desmoglein-1-alpha	3	1.2	2.43E-02
Ablim2	Actin-binding LIM protein 2 (Fragment)	1	1.2	4.10E-03
Acot2	Acyl-coenzyme A thioesterase 2, mitochondrial	8	1.1	2.86E-04
Hsp90ab1	Heat shock protein HSP 90-beta	2	1.0	1.53E-02
Try10	Trypsin 10	2	1.0	2.14E-03
Vwa8	von Willebrand factor A domain-containing protein 8	22	1.0	8.68E-04
**Gene Name**	**Protein Name**	**# Peptides**	**Enrichment (log_2 Fold change HFHSD vs SD)**	**p Value**
Hmgcl	Hydroxymethylglutaryl-CoA lyase, mitochondrial	4	-2.7	6.02E-03
Ghitm	Growth hormone-inducible transmembrane protein	1	-2.2	7.82E-07
Hk1	Hexokinase-1	8	-1.8	1.33E-02
Bdh1	D-beta-hydroxybutyrate dehydrogenase, mitochondrial	14	-1.6	1.78E-04
Ndufa9	NADH dehydrogenase [ubiquinone] 1 alpha subcomplex subunit 9, mitochondrial	12	-1.6	4.41E-04
Eno3	Beta-enolase	4	-1.6	4.06E-04
Jph2	Junctophilin-2	3	-1.3	3.24E-02
Rab1a	RAB1A, member RAS oncogene family	5	-1.2	2.54E-03
Bckdhb	2-oxoisovalerate dehydrogenase subunit beta, mitochondrial	3	-1.2	1.98E-02
Tomm40	Mitochondrial import receptor subunit TOM40 homolog	3	-1.0	1.60E-02
Pccb	Propionyl-CoA carboxylase beta chain, mitochondrial	9	-1.0	7.32E-03
Bckdha	2-oxoisovalerate dehydrogenase subunit alpha	9	-1.0	3.87E-03
Slc25a11	Mitochondrial 2-oxoglutarate/malate carrier protein	9	-1.0	3.17E-03
Flna	Filamin-A	5	-0.9	3.13E-02
Ptpn5	Tyrosine-protein phosphatase non-receptor type 5	1	-0.8	2.76E-03

Additional unaltered key MAM proteins				
**Gene Name**	**Protein Name**	**# Peptides**	**Enrichment (log_2 Fold change HFHSD vs SD)**	**p Value**
Vdac1	Voltage-dependent anion-selective channel protein 1	15	0	9.98E-01
Mfn1	Mitofusin-1	5	0.1	8.63E-01
RyR2	Ryanodine receptor 2	31	0.2	4.20E-01
Hspa9: Grp75	Stress-70 protein, mitochondrial	19	0	8.57E-01
Atp2a2: Serca2	Sarcoplasmic/endoplasmic reticulum calcium ATPase 2	45	0.5	9.52E-02

Supplementary Table 1: Significantly up- and down-regulated cardiac MAM proteins in the HFHSD versus SD mouse.

	Upregulated in HFHSD	Downregulated in HFHSD
**Cellular cation homeostasis**		Hk1<br>Jph2
**Signal transduction**	Ndrg2	Rab1a<br>Hk1
**Protein and ion transport**		Rab1a<br>Tomm40<br>Slc25a11
**Biological regulation**	Ndrg2<br>Jup<br>Hsp90ab1	Hk1<br>Bdh1<br>Ndufa9<br>Rab1a<br>Bckdhb
**Organelle organization**	Dsp<br>Tpm1	Rab1a<br>Tomm40
**Cellular component assembly**	Ablim2	Jph2<br>Rab1a<br>Flna
**Response to stress**	Dsp<br>Hsp90ab1<br>Fgfr1op2	Ghitm<br>Hk1
**ATP metabolic process**	Vwa8	Hk1<br>Eno3<br>Pccb
**Nitrogen compound metabolic process**	Jup<br>Acot2<br>Try10	Hmgcl<br>Hk1<br>Rab1a<br>Bckdha
**Lipid metabolic process**	Fabp4<br>Acot2<br>Fabp5<br>Hsd17b4	Hmgcl
**Protein metabolic process**	Try10	Rab1a<br>Ptpn5

Supplementary Table 2: List of the proteins involved in the major biological processes significantly up- and down-regulated in cardiac HFHSD MAM.

Supplementary Table 3 Ca^{2+} transient characteristics in SD and HFHSD cardiomyocytes, under 0.5 Hz and 1 Hz field stimulation

Field stimulation frequency	Group	n	F/F_0	TTP (s)	$t_{1/2}$ (s)	TPB (s)
0.5 Hz	SD	45	0.91 [0.60, 1.50]	0.17 [0.13, 0.23]	0.49 [0.38, 0.72]	1.86 [1.75, 1.88]
	HFHSD	39	0.91 [0.38, 1.38]	0.18 [0.15, 0.31]	0.51 [0.27, 0.79]	1.84 [1.71, 1.87]
1 Hz	SD	56	0.83 [0.46, 1.25]	0.11 [0.09, 0.12]	0.34 [0.29, 0.40]	0.91 [0.89, 0.92]
	HFHSD	49	0.80 [0.42, 1.11]	0.11 [0.09, 0.19]	0.33 [0.23, 0.41]	0.90 [0.85, 0.92]

Data are displayed as median [Interquartile range]. F/F_0: peak amplitude; TTP: time to peak; $t_{1/2}$: half-time; TPB: time peak to basal. N=4 mice/group.

SUPPLEMENTARY BIBLIOGRAPHY

1. Bartok A, Weaver D, Golenar T, Nichtova Z, Katona M, Bansaghi S, Alzayady KJ, Thomas VK, Ando H, Mikoshiba K, Joseph SK, Yule DI, Csordas G, Hajnoczky G (2019) IP3 receptor isoforms differently regulate ER-mitochondrial contacts and local calcium transfer. Nat Commun 10:3726 doi:10.1038/s41467-019-11646-3
2. Chouabe C, Espinosa L, Megas P, Chakir A, Rougier O, Freminet A, Bonvallet R (1997) Reduction of I(Ca,L) and I(to1) density in hypertrophied right ventricular cells by simulated high altitude in adult rats. J Mol Cell Cardiol 29:193-206 doi:10.1006/jmcc.1996.0264
3. Espinosa L, Chouabe C, Morales A, Lachuer J, Georges B, Fatemi M, Terrenoire C, Tourneur Y, Bonvallet R (2000) Increased sodium-calcium exchange current in right ventricular cell hypertrophy induced by simulated high altitude in adult rats. J Mol Cell Cardiol 32:639-653 doi:10.1006/jmcc.2000.1106
4. O'Connell TD, Rodrigo MC, Simpson PC (2007) Isolation and culture of adult mouse cardiac myocytes. Methods Mol Biol 357:271-296 doi:10.1385/1-59745-214-9:271
5. Paccalet A, Tessier N, Paillard M, Paita L, Gomez L, Gallo-Bona N, Chouabe C, Leon C, Badawi S, Harhous Z, Ovize M, Crola Da Silva C (2020) An innovative sequence of hypoxia-reoxygenation on adult mouse cardiomyocytes in suspension to perform multilabeling analysis by flow cytometry. Am J Physiol Cell Physiol 318:C439-C447 doi:10.1152/ajpcell.00393.2019
6. Paillard M, Csordas G, Szanda G, Golenar T, Debattisti V, Bartok A, Wang N, Moffat C, Seifert EL, Spat A, Hajnoczky G (2017) Tissue-Specific Mitochondrial Decoding of Cytoplasmic Ca(2+) Signals Is Controlled by the Stoichiometry of MICU1/2 and MCU. Cell Rep 18:2291-2300 doi:10.1016/j.celrep.2017.02.032
7. Paillard M, Tubbs E, Thiebaut PA, Gomez L, Fauconnier J, Da Silva CC, Teixeira G, Mewton N, Belaidi E, Durand A, Abrial M, Lacampagne A, Rieusset J, Ovize M (2013) Depressing mitochondria-reticulum interactions protects cardiomyocytes from lethal hypoxia-reoxygenation injury. Circulation 128:1555-1565 doi:10.1161/CIRCULATIONAHA.113.001225
