# Supplementary material for: Reduced reticulum–mitochondria Ca2+ transfer is an early and reversible trigger of mitochondrial dysfunctions in diabetic cardiomyopathy
Source: Basic Res Cardiol. 2020 Nov 30;115(6):74. doi: 10.1007/s00395-020-00835-7 (PMC7704523; doi:10.1007/s00395-020-00835-7)

Full unedited gel for Figure 1c

Phosphorylated AKT With and without insulin stimulation

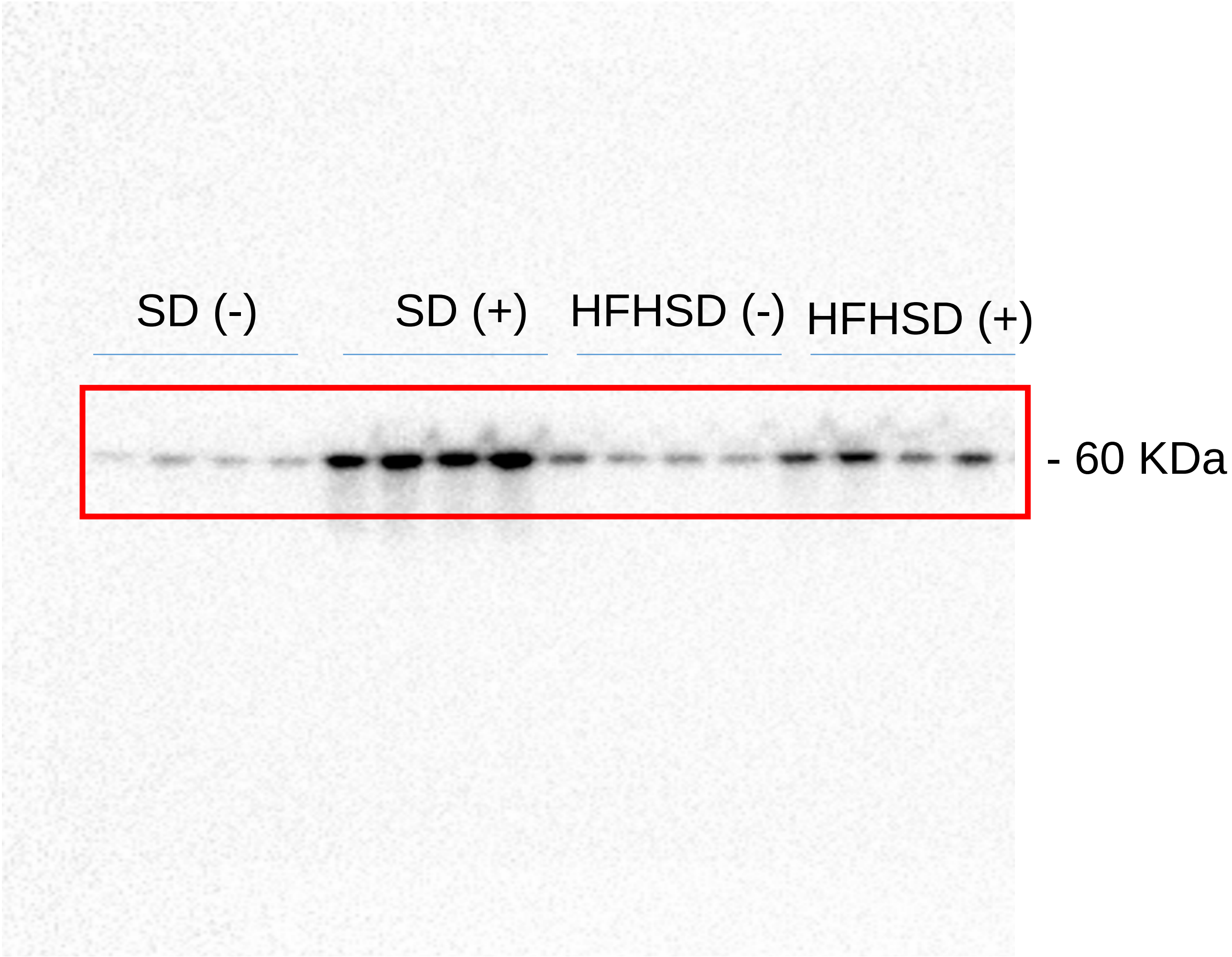

Full unedited gel for Figure 1c

Total AKT
With and without
insulin stimulation

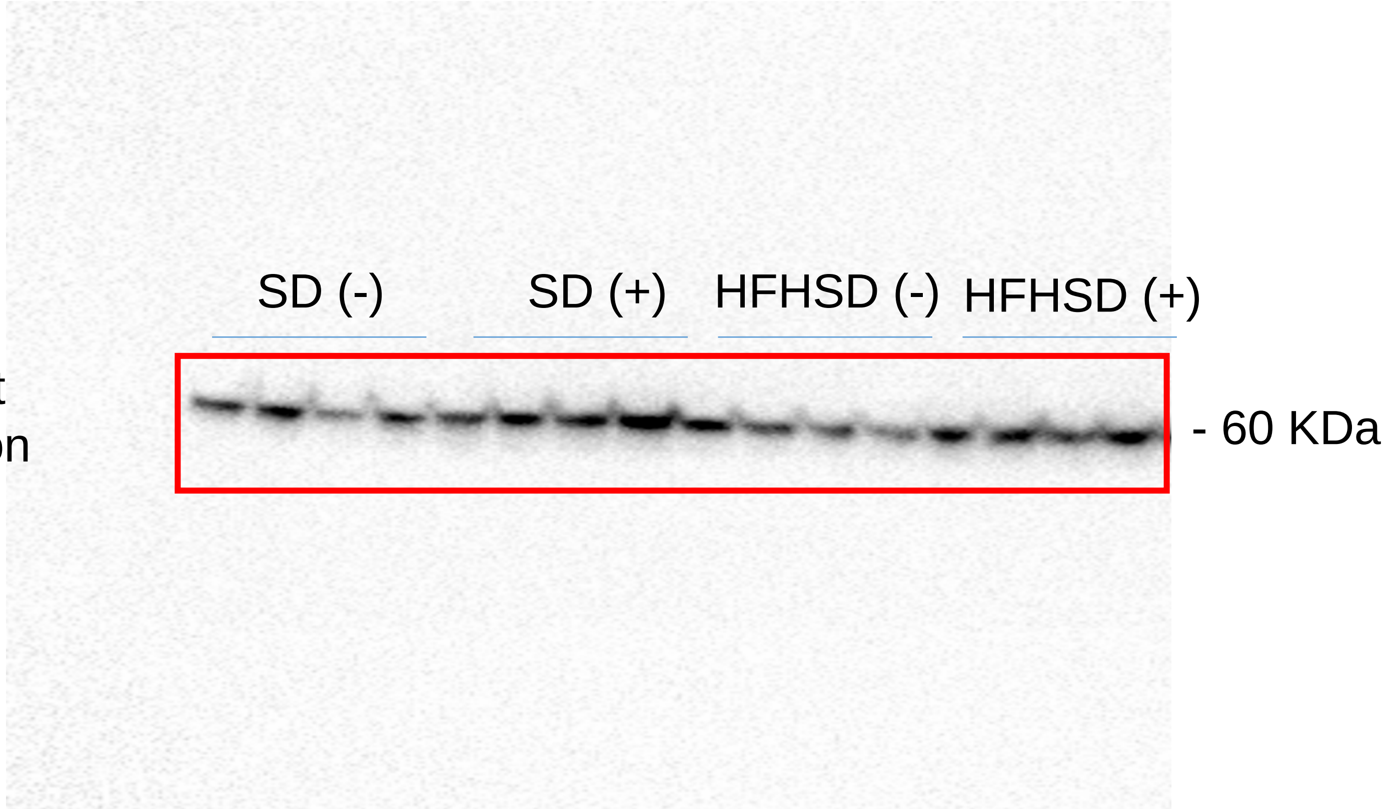

Full unedited gel for Figure 2h

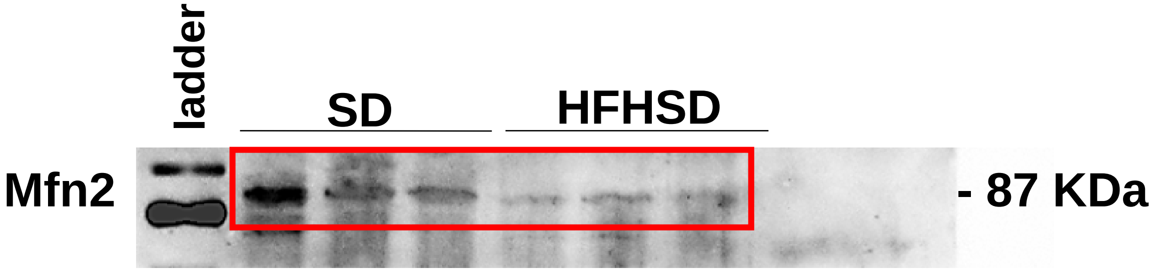

Full unedited gel for Figure 2h

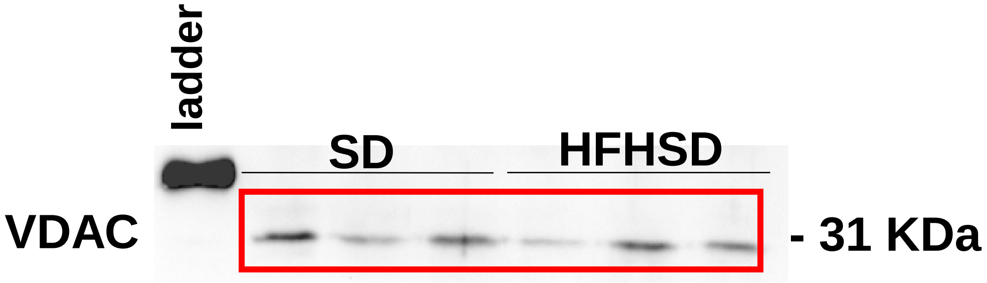

Full unedited gel for Figure 3b

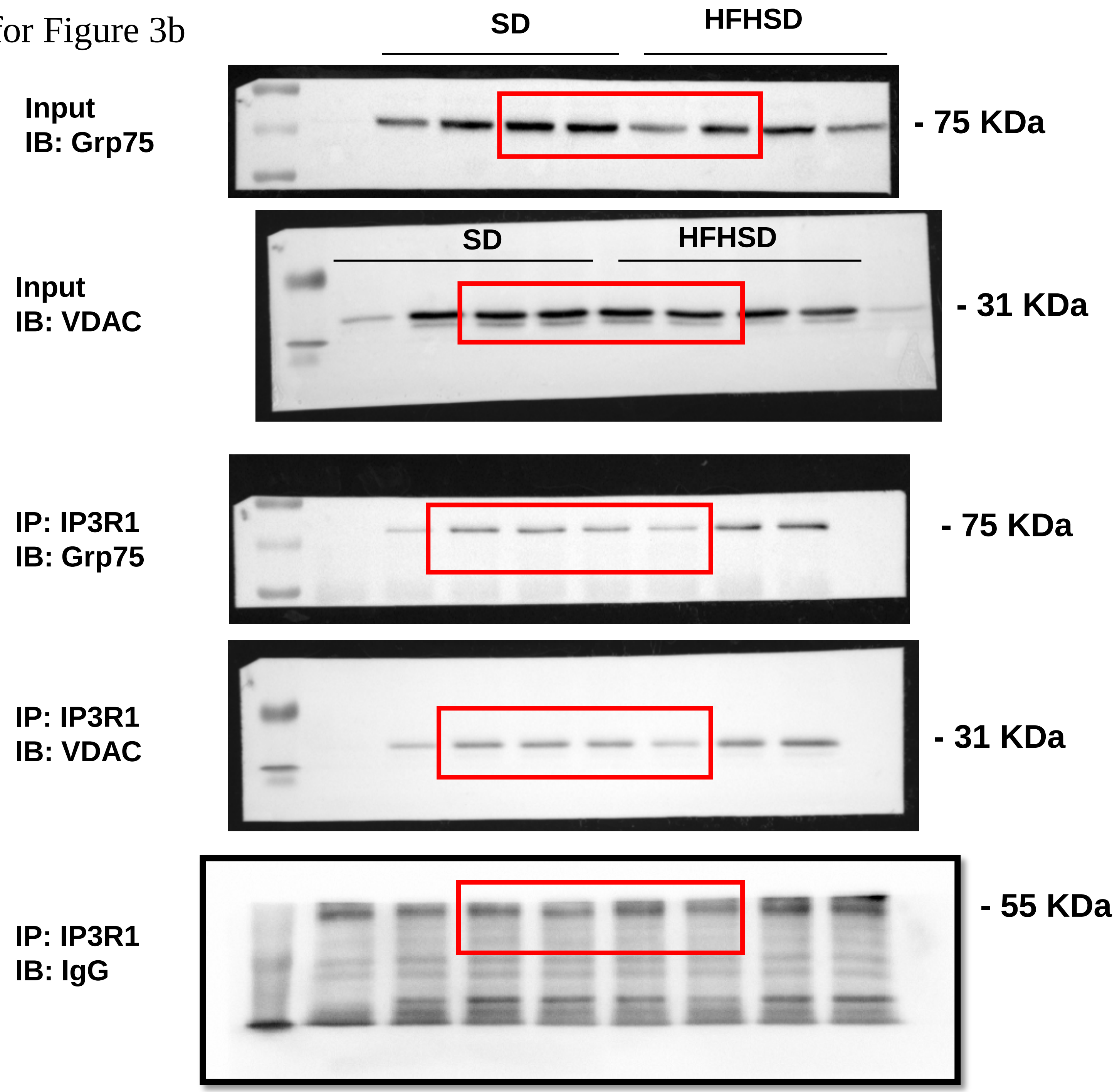

Full unedited gel for Figure 3h

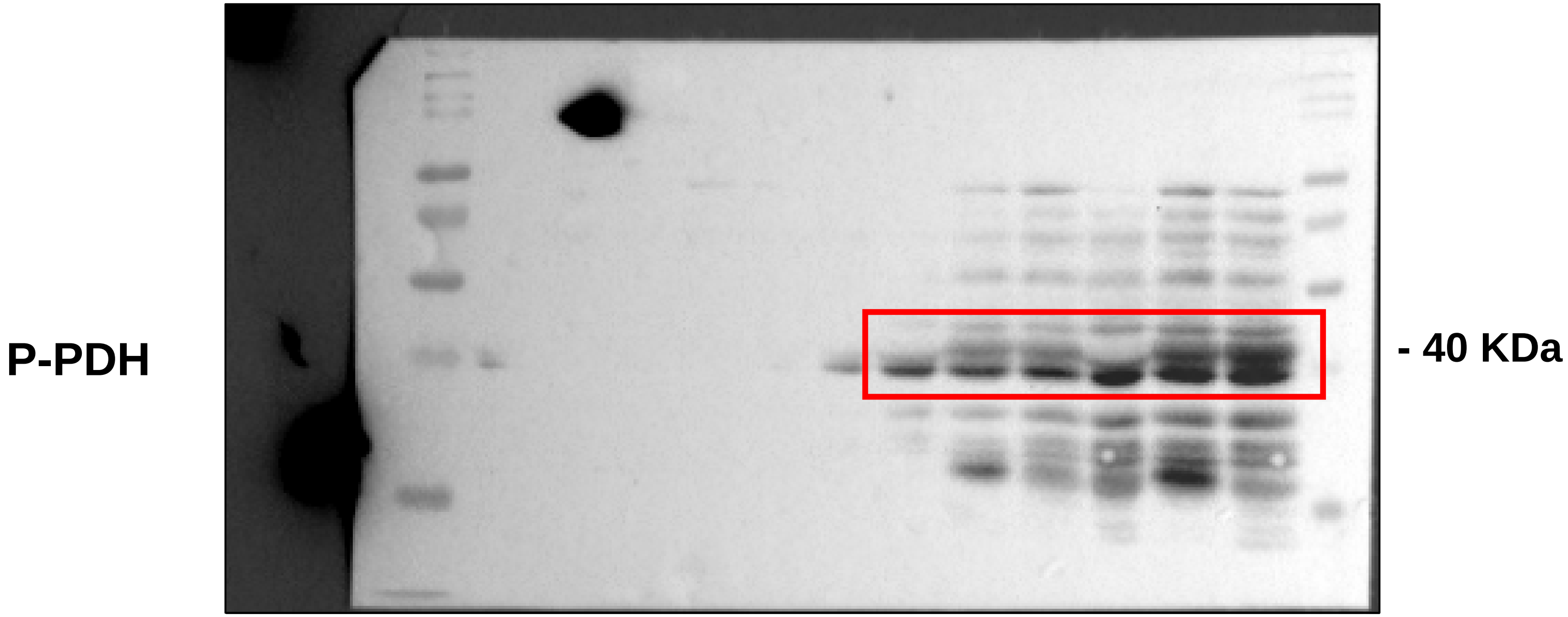

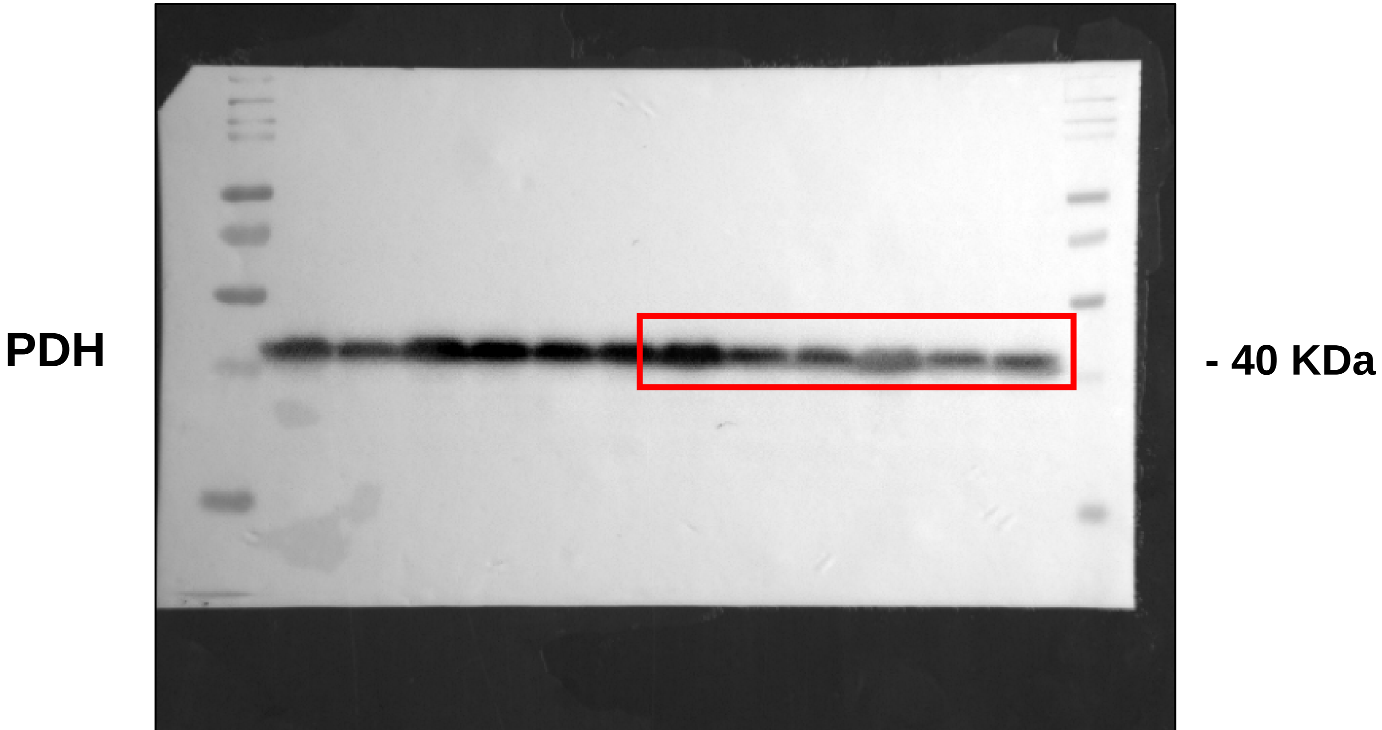

Full unedited gel for
Figure 5a

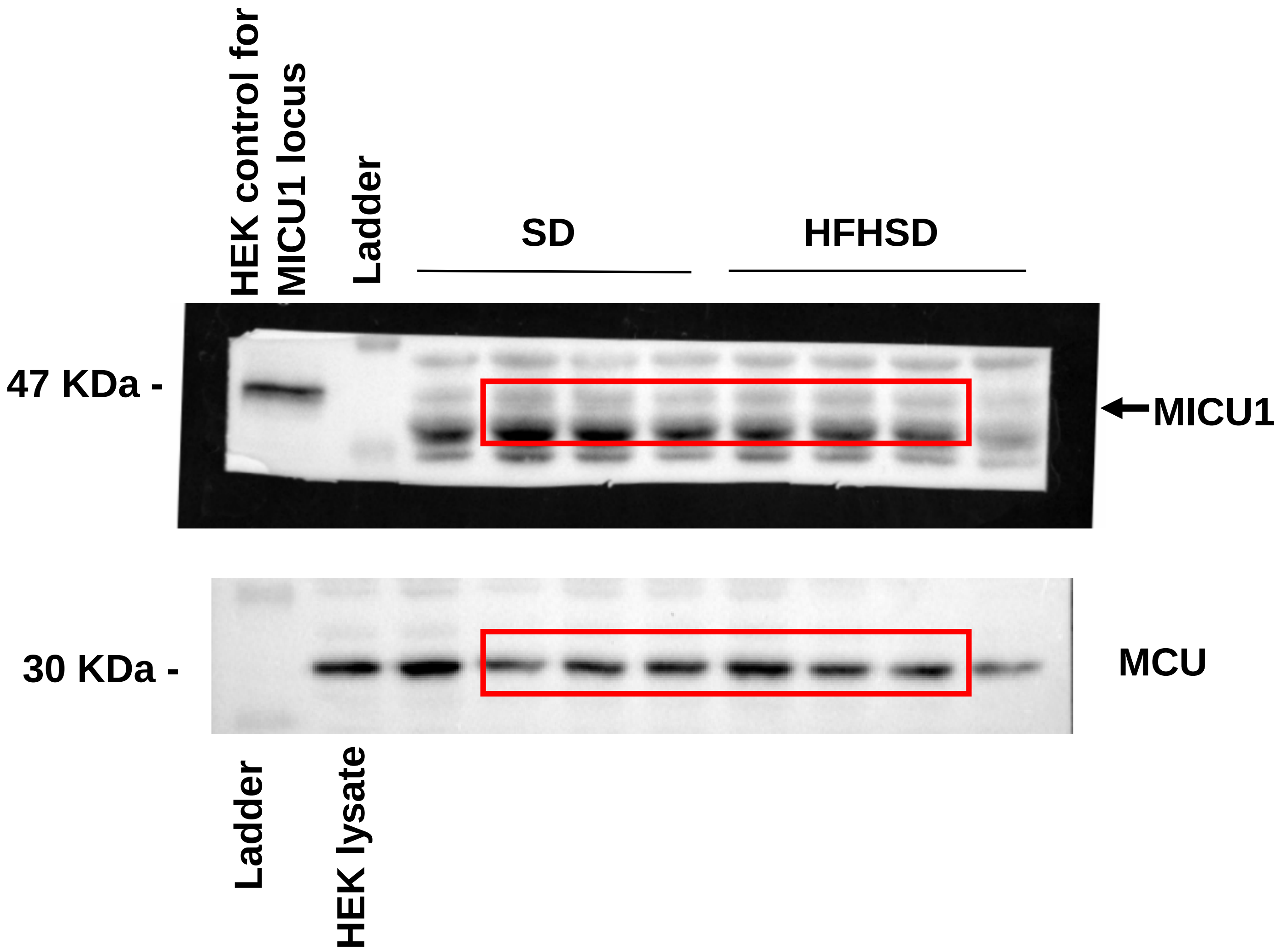

Full unedited gel for
Figure 6h

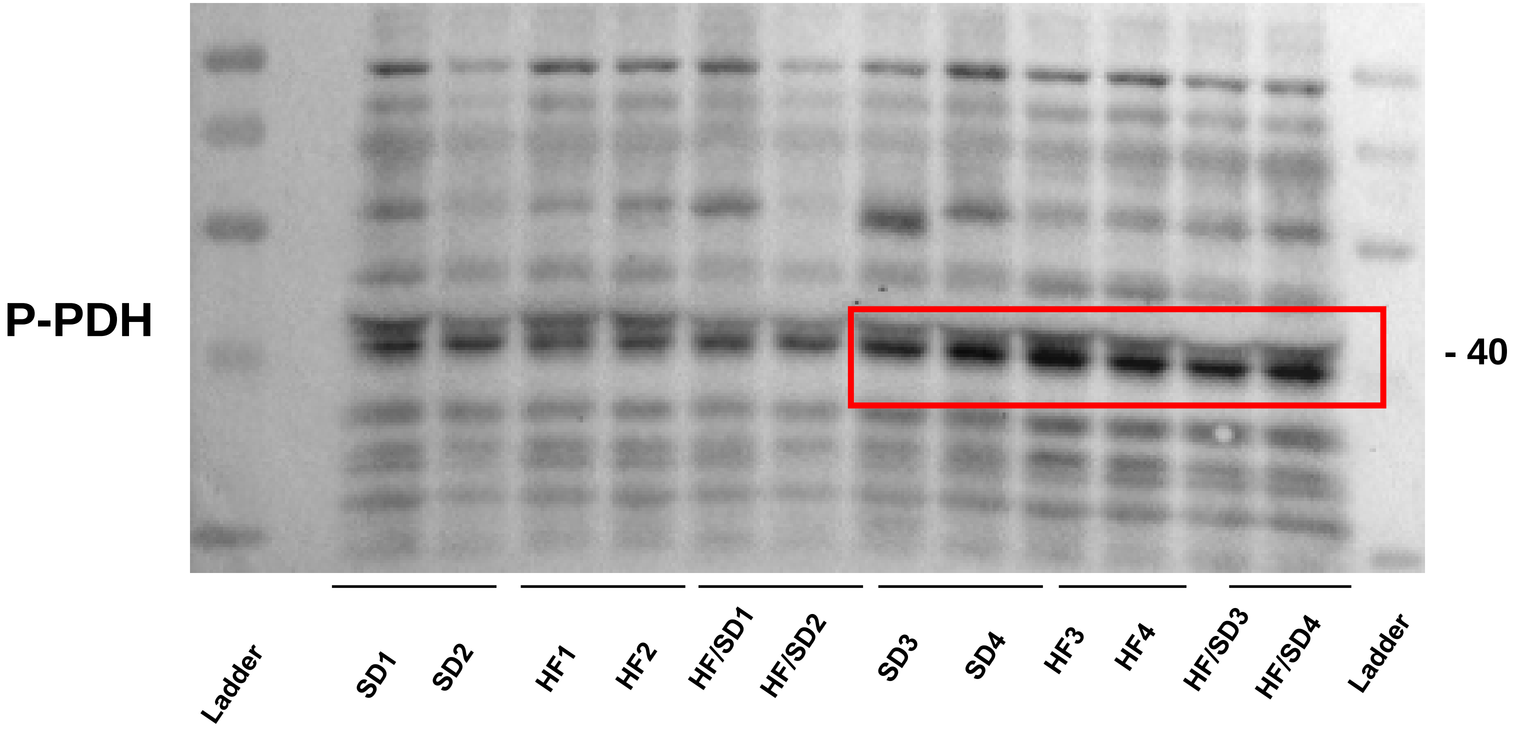

Full unedited gel for
Figure 6h

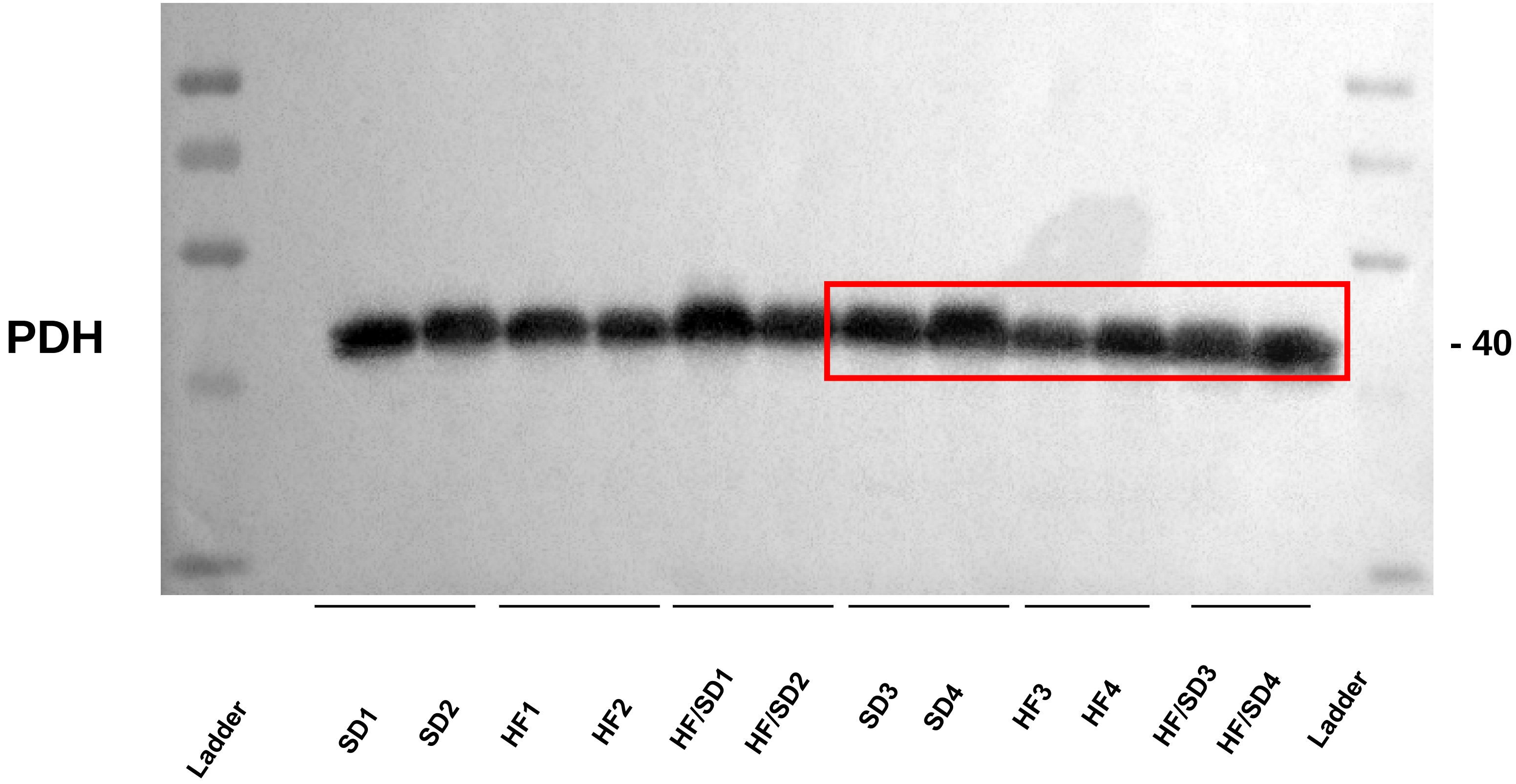

Full unedited gel for
Supplemental Figure 3d

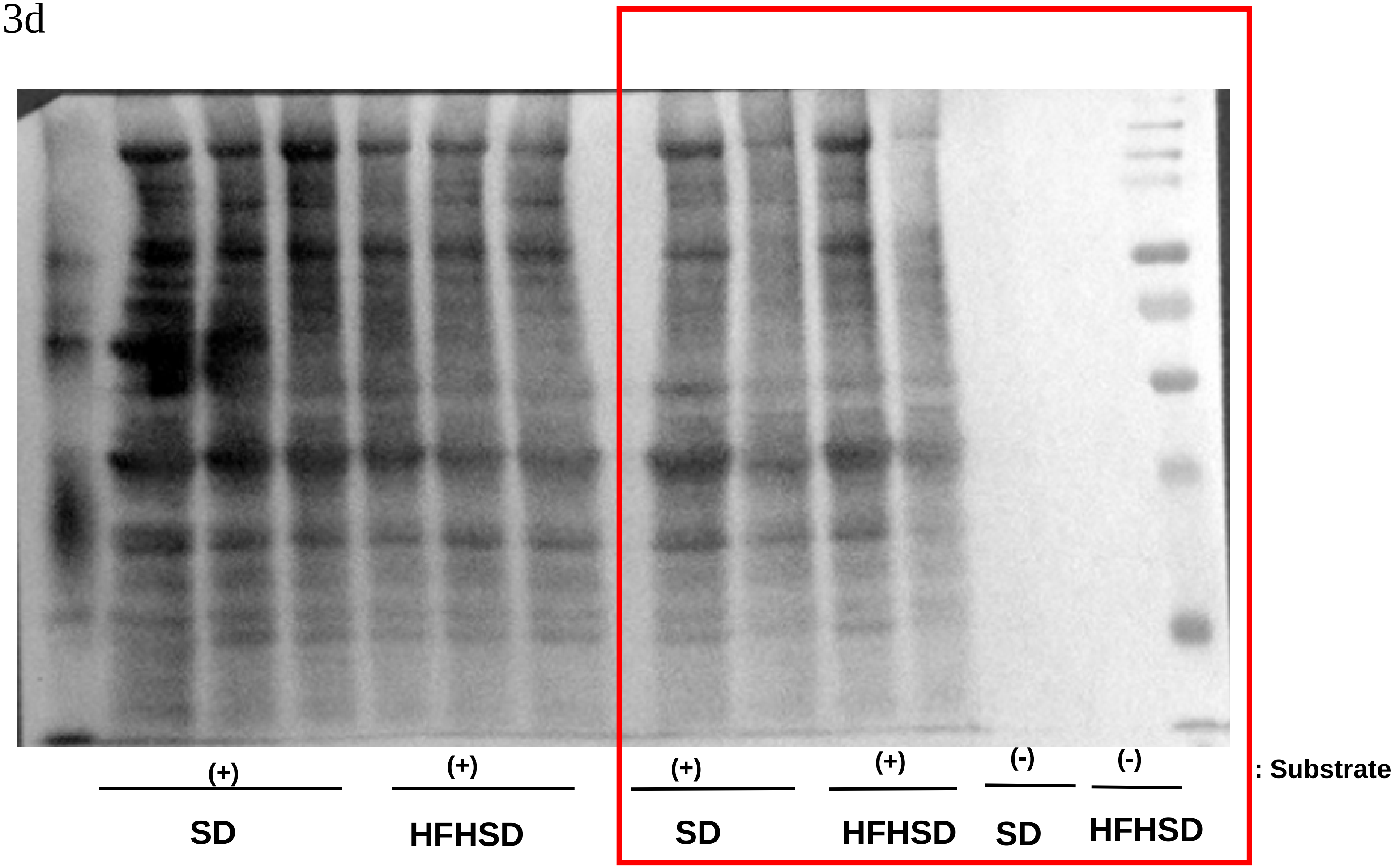

Supplement: Supplementary file 2 — Supplementary file2 (PDF 1890 kb) [file 395_2020_835_MOESM2_ESM.pdf]
